# Supplementary material for: Transcriptional landscape of direct reprogramming toward the hematopoietic lineage
Source: iScience. 2026 Aug 25;29(9):117266. doi: 10.1016/j.isci.2026.117266 (PMC13543898; doi:10.1016/j.isci.2026.117266)
Supplement: Supplementary file 1 [file mmc1.pdf]

iScience, Volume *29*

Supplemental information

Transcriptional landscape of direct reprogramming toward the hematopoietic lineage

Jillian Cwycyshyn, Cooper Stansbury, Sarah Golts, Hyunsu Lee, Joshua Pickard, Walter Meixner, Indika Rajapakse, and Lindsey A. Muir

Supplemental Material

Supplementary Figures

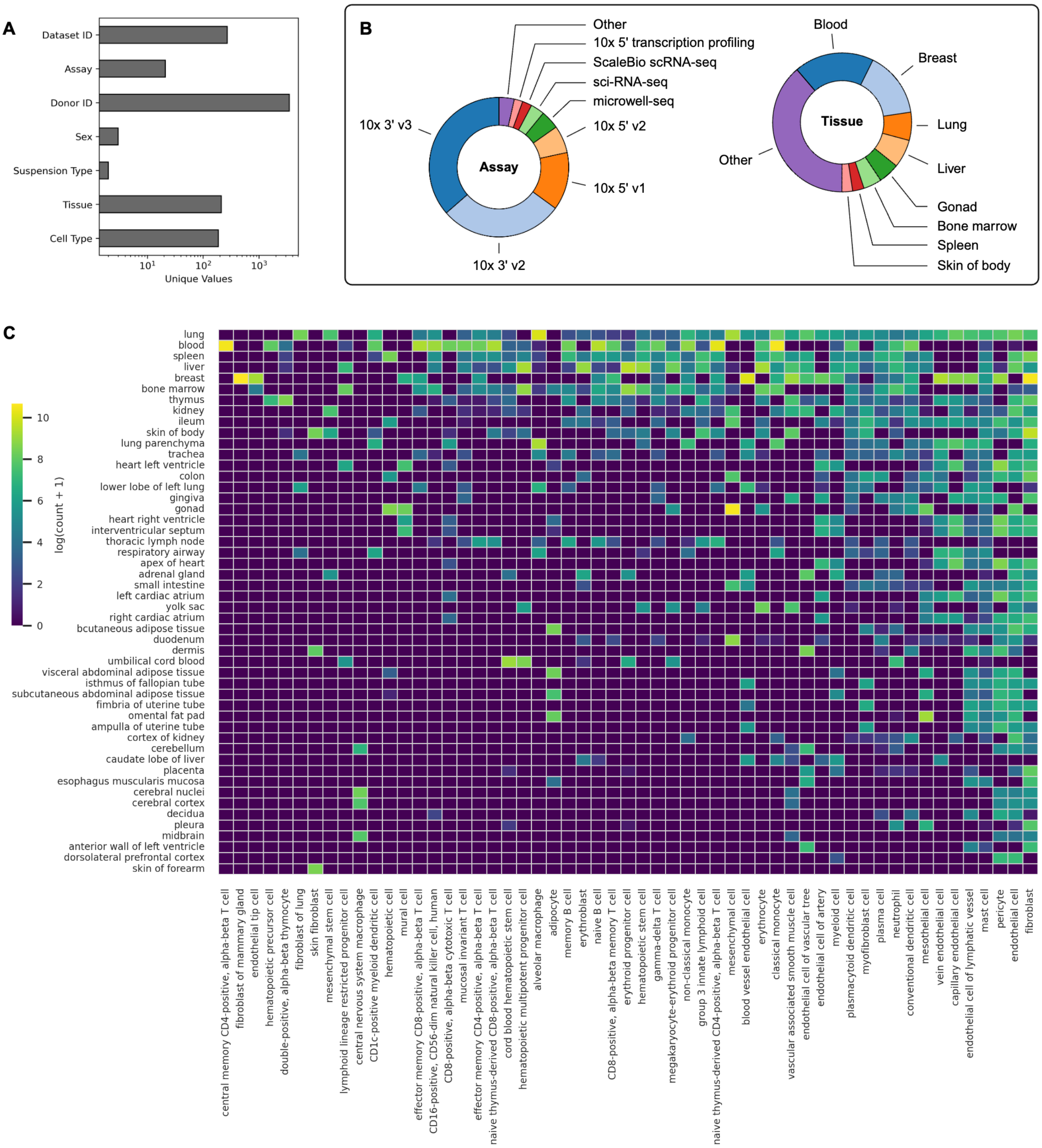

Figure S1: **Overview of tissue, cell type, and metadata composition in the reference atlas, related to Figure 1. (A)** Number of unique values per metadata field (log scale). **(B)** Distribution of tissue sources and assay modalities. **(D)** Heatmap showing log-transformed counts of cells ($\log(\text{count} + 1)$) for the most abundant tissues and cell types across the dataset. Tissues (rows) and cell types (columns) were selected to collectively represent 90% of the total counts in each category. Created in BioRender. Cwycyshyn, J. (2026) https://BioRender.com/6btz88i.

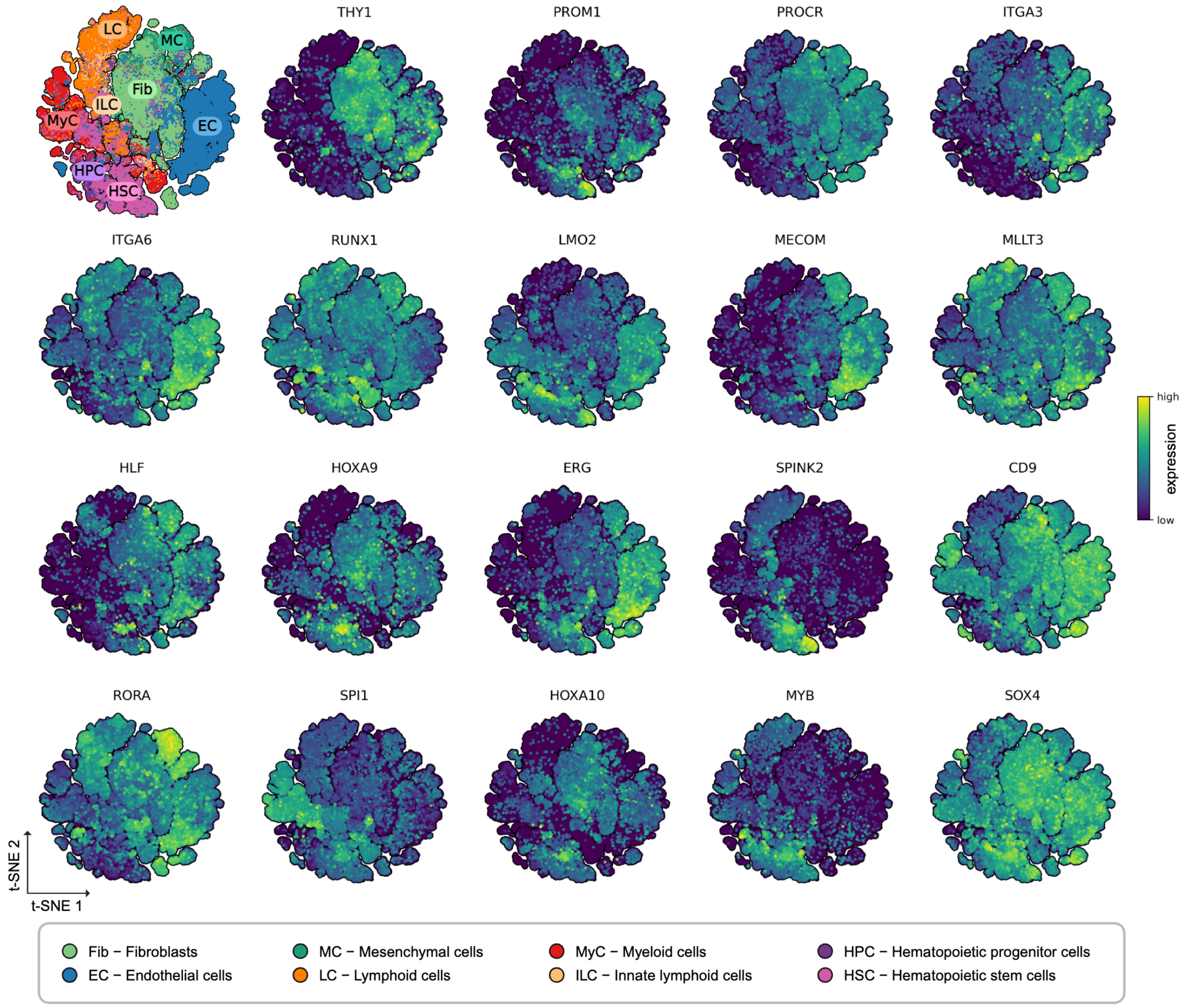

Figure S2: **Gene expression across reference atlas, related to Figure 1.** Expression patterns of selected genes across all cell types, visualized in a shared low-dimensional (t-SNE) embedding. Each panel shows log-normalized expression of a single gene. Created in BioRender. Cwycyshyn, J. (2026) https://BioRender.com/httoooo.

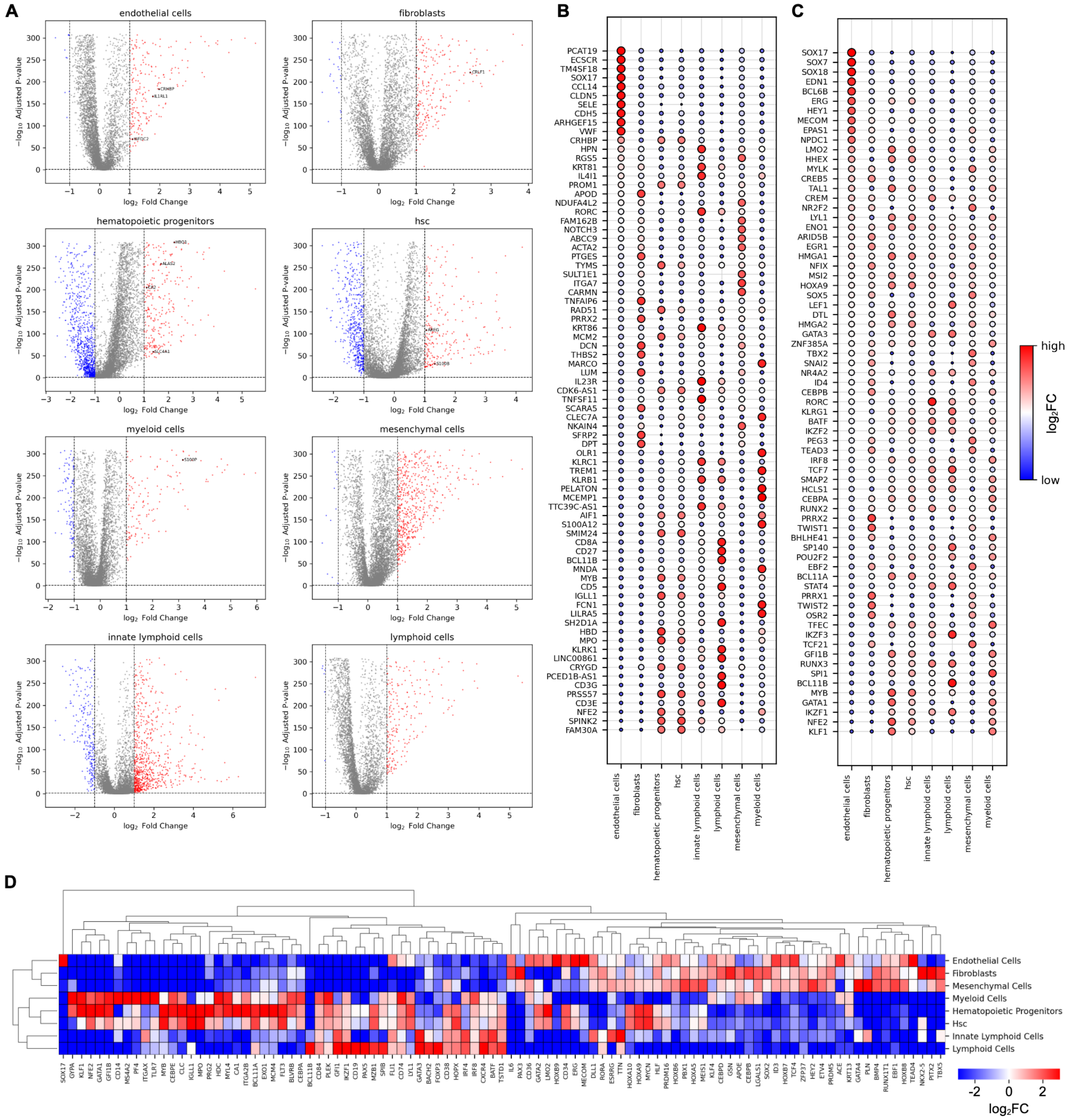

Figure S3: **Differential expression of major cellular compartments, related to Figure 1. (A)** Volcano plots for DEGs for each group (Wilcoxon rank-sum test). Genes with adjusted p-value < 0.05 and absolute $\log_2$ fold change > 1 were considered significant (red: upregulated, blue: downregulated). Dashed lines indicate the significance thresholds. Marker genes derived from Tabula Sapiens[1]. **(B)** The top 10 DEGs per group, filtered to include only genes expressed in at least 15% of cells, with color and size indicating $\log_2$ fold change. **(C)** The top 10 differentially expressed TFs per group, filtered to include only genes expressed in at least 15% of cells, with color and size indicating $\log_2$ fold change. **(D)** Differential expression of literature-curated genes across cell types. Genes filtered significant differential expression (adjusted p-value < 0.05) and high variability (standard deviation > 1.5) in $\log_2$ fold change. The heatmap shows average expression changes across annotated cell types, clustered by gene and cell identity. Created in BioRender. Cwycyshyn, J. (2026) https://BioRender.com/uqhsjuf.

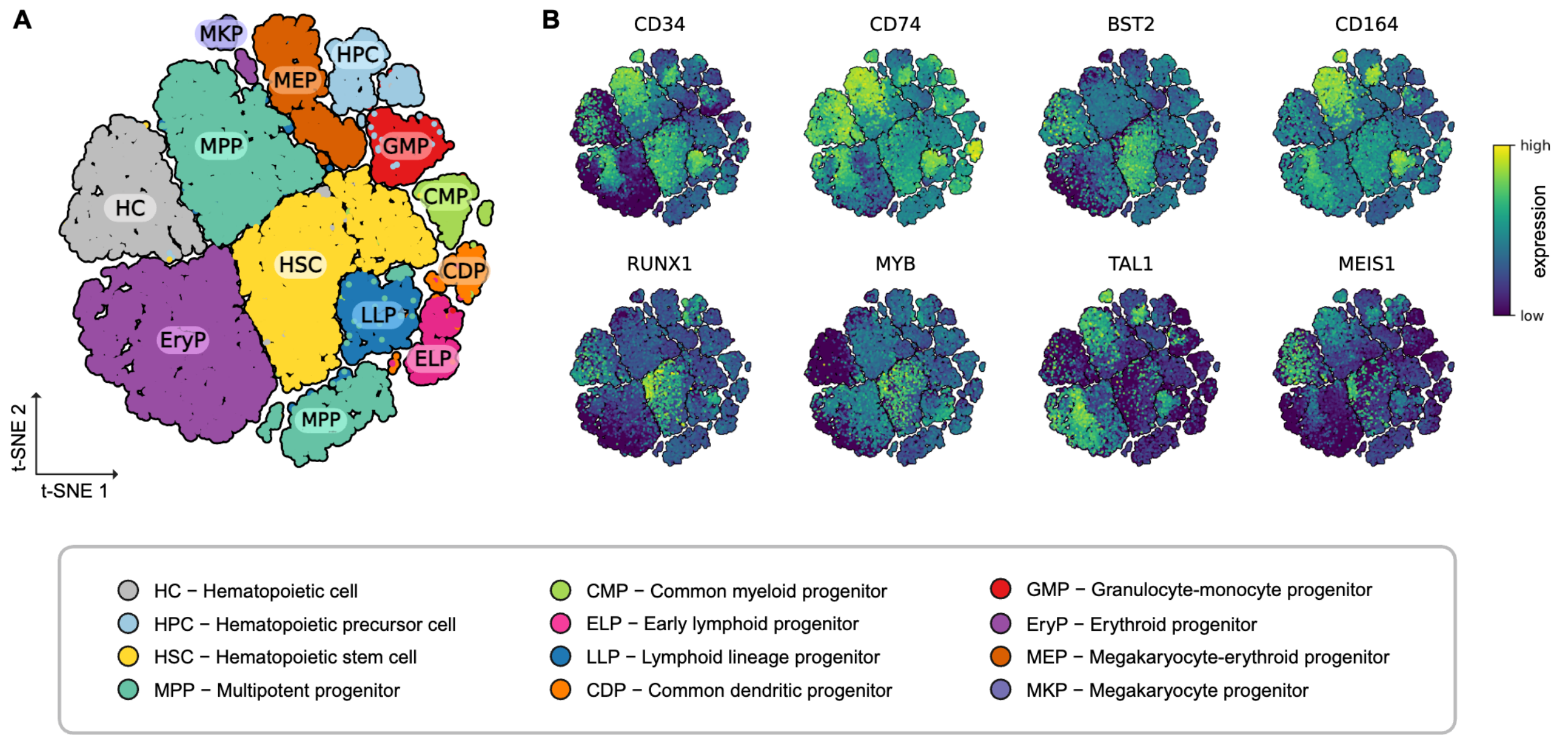

Figure S4: **Marker gene expression in hematopoietic cells from the reference atlas, related to Figure 1. (A)** t-SNE of major hematopoietic cell types. **(B)** Expression of marker genes on coordinates from (A). Created in BioRender. Cwycyshyn, J. (2026) https://BioRender.com/uujcpsn.

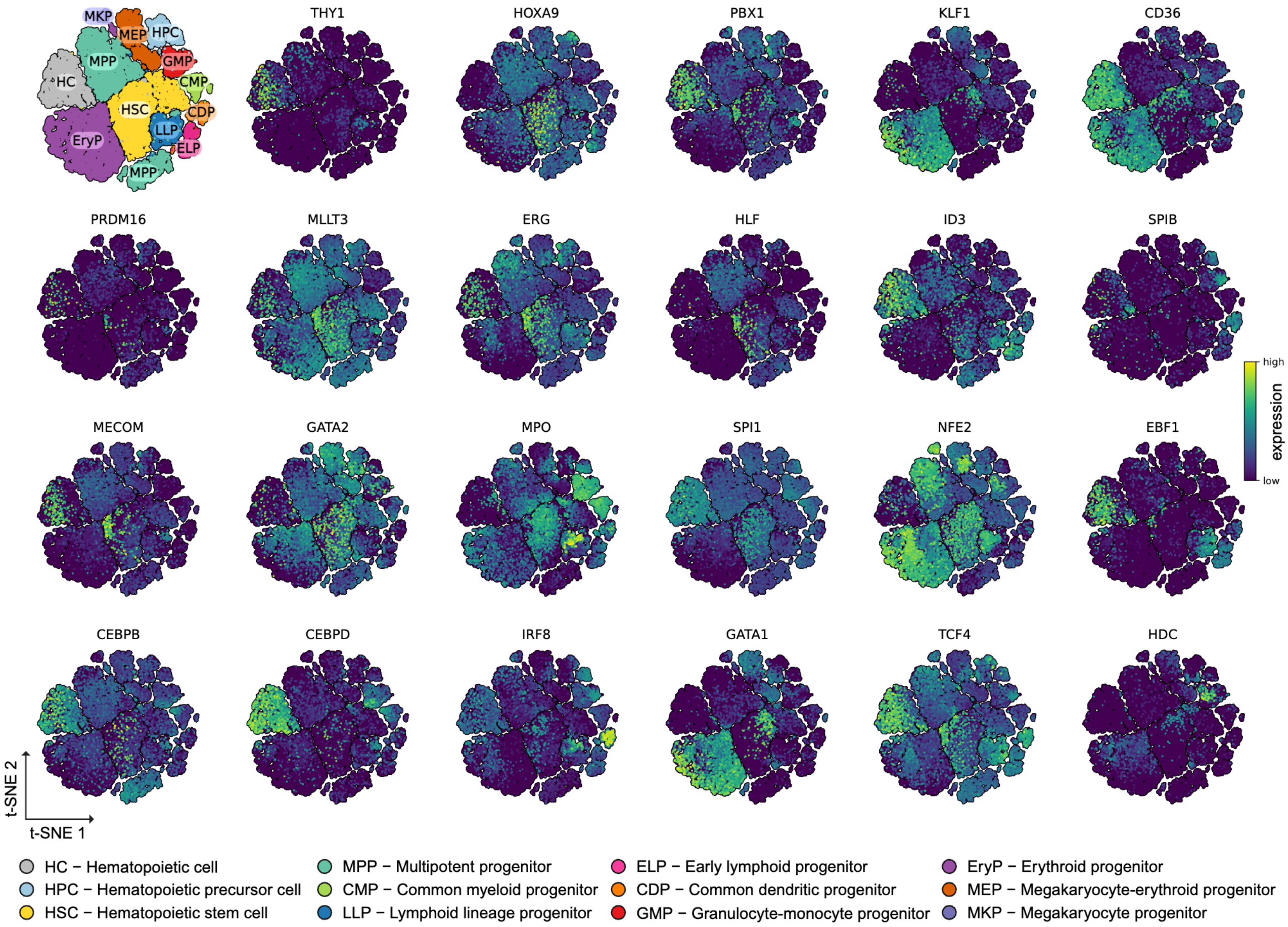

Figure S5: **Gene expression across hematopoietic populations in the reference atlas, related to Figure 1.** Expression patterns of selected genes across hematopoietic cell types, visualized in a shared low-dimensional (t-SNE) embedding. The top left t-SNE plot is colored by cell type. All other t-SNE plots show log-normalized expression of a single gene. Created in BioRender. Cwycyshyn, J. (2026) https://BioRender.com/1r5rvub.

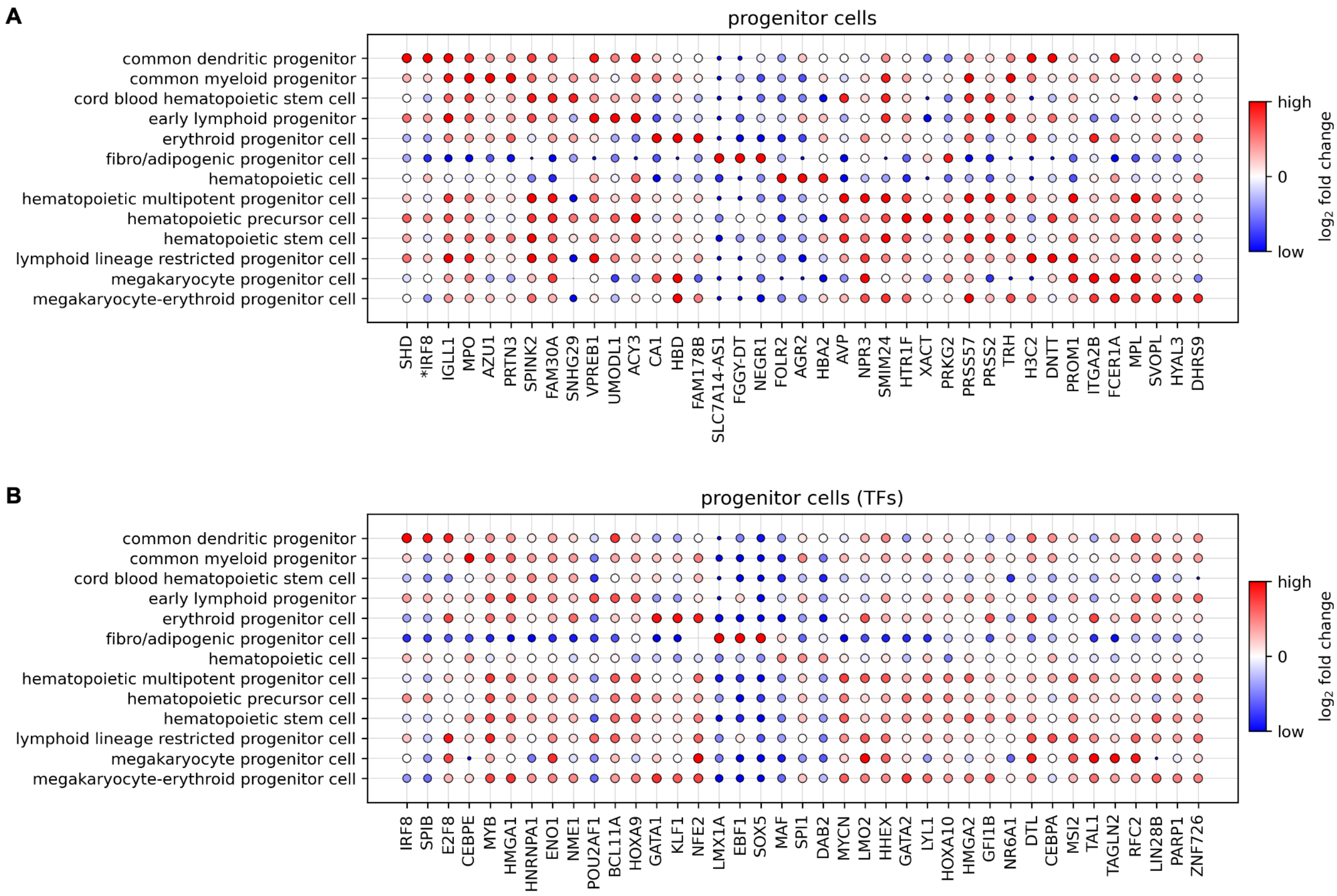

Figure S6: **Differentially expressed genes across hematopoietic progenitor subtypes, related to Figure 1.** The top 3 unique **(A)** DEGs and **(B)** differentially expressed TFs for each cell type. The size and color of each dot represents $\log_2$ fold change. Asterisks in (A) denote TFs. Created in BioRender. Cwycyshyn, J. (2026) https://BioRender.com/7idms0y.

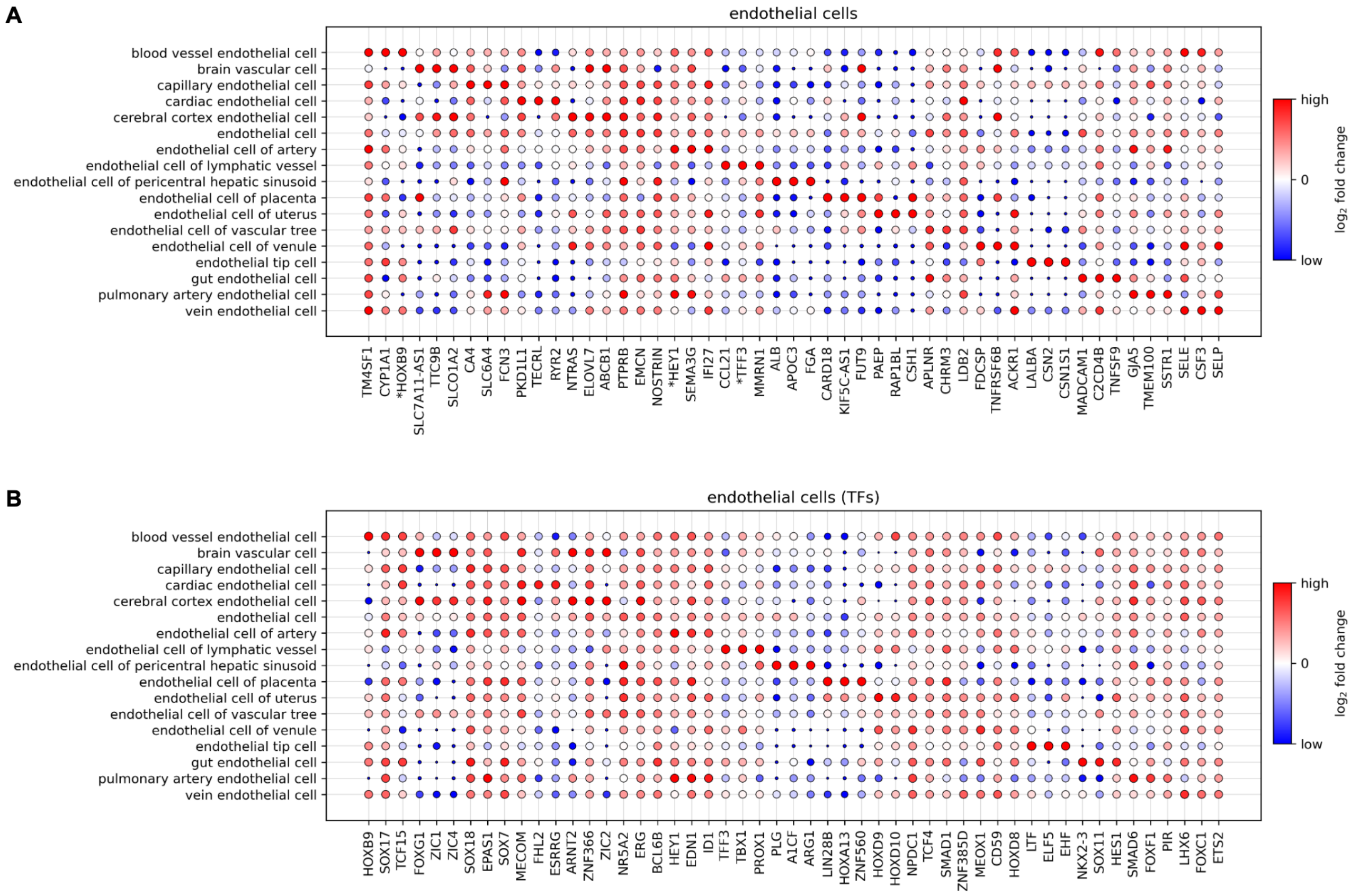

Figure S7: **Differentially expressed genes across endothelial cell subtypes, related to Figure 1.** The top 3 unique **(A)** DEGs and **(B)** differentially expressed TFs for each cell type. The size and color of each dot represents $\log_2$ fold change. Asterisks in (A) denote TFs. Created in BioRender. Cwycyshyn, J. (2026) https://BioRender.com/v8r0b1x.

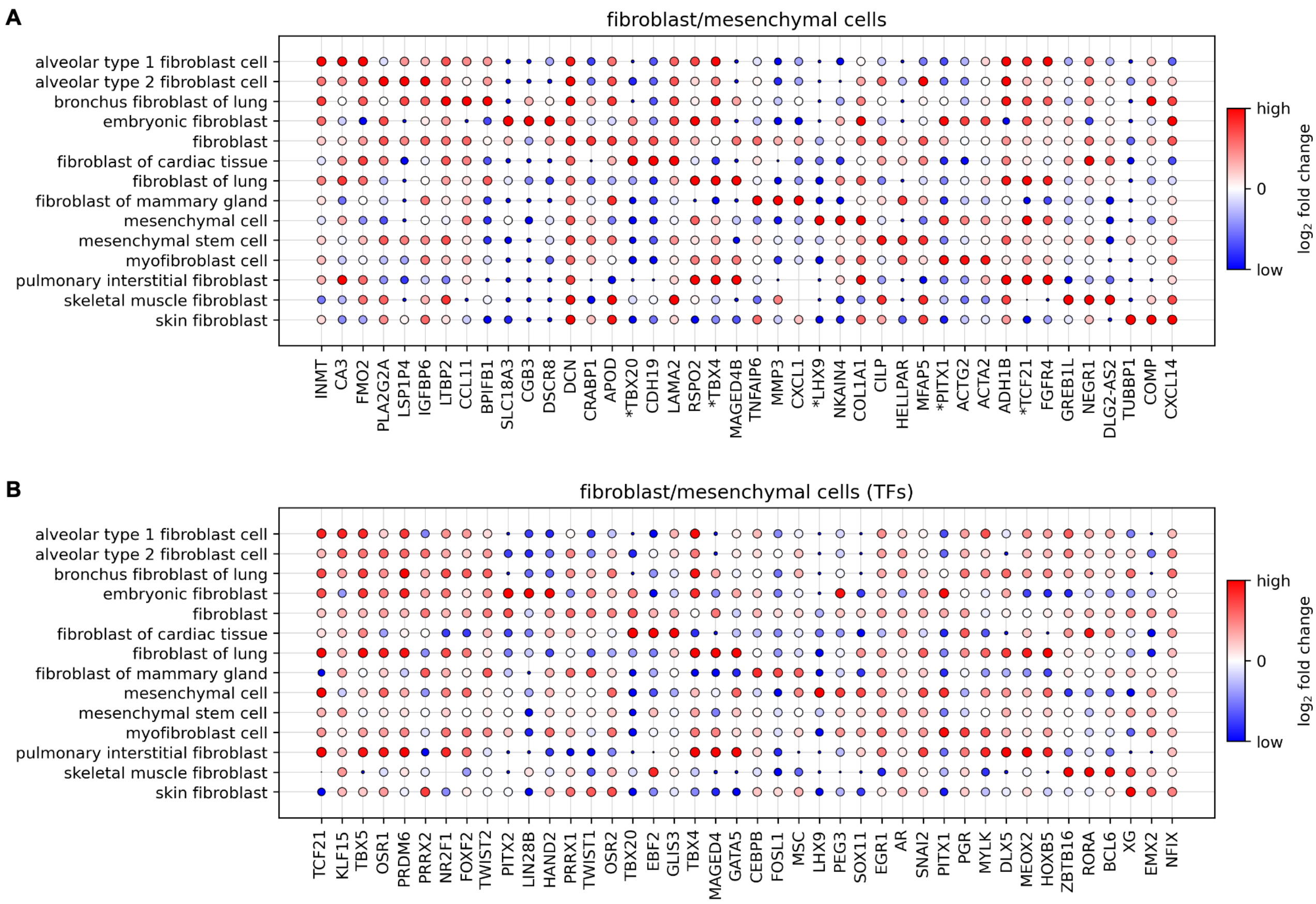

Figure S8: **Differentially expressed genes across fibroblast and mesenchymal subtypes, related to Figure 1.** The top 3 unique **(A)** DEGs and **(B)** differentially expressed TFs for each cell type. The size and color of each dot represents $\log_2$ fold change. Asterisks in (A) denote TFs. Created in BioRender. Cwycyshyn, J. (2026) https://BioRender.com/ixbxvpj.

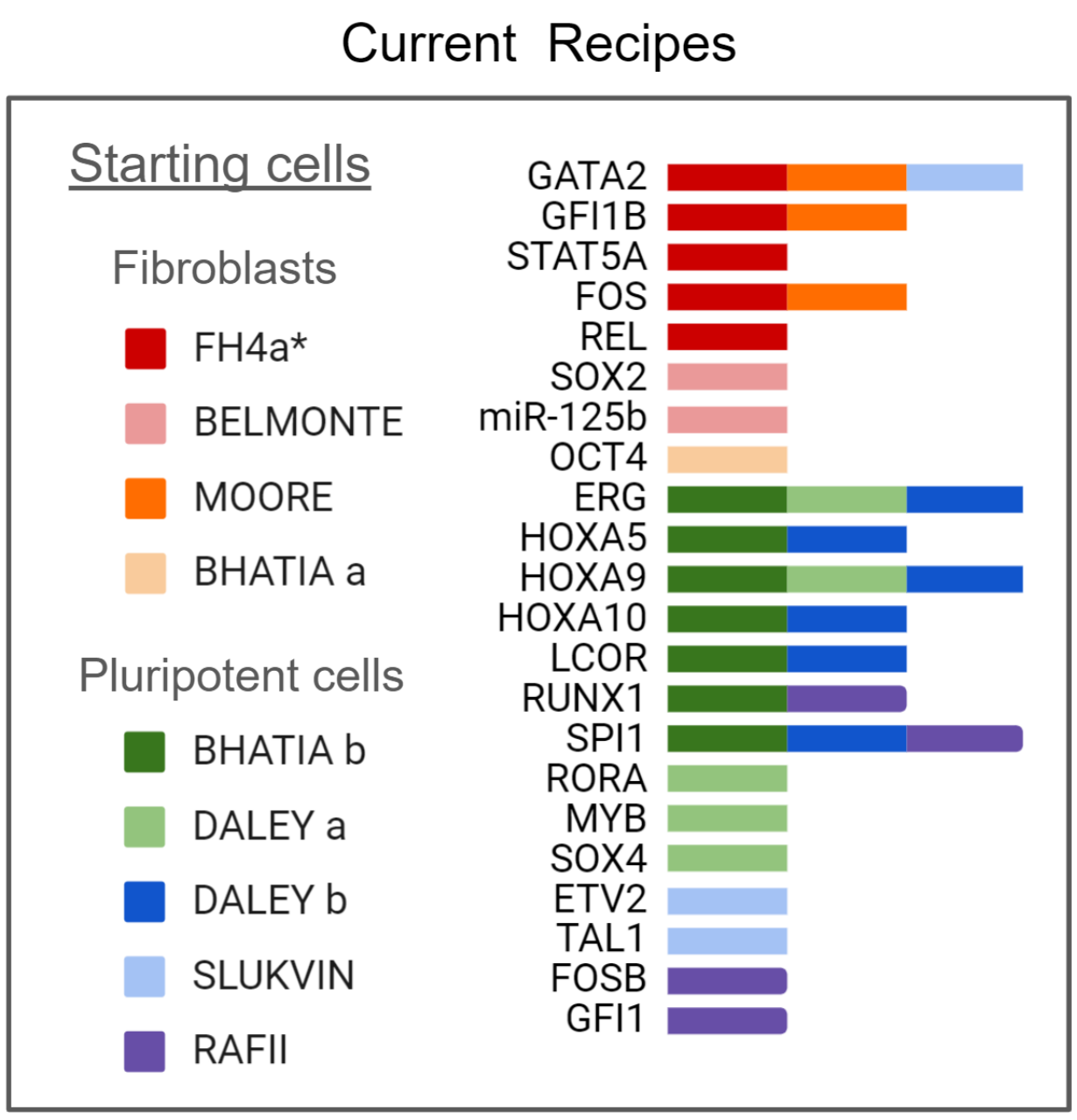

Figure S9: **Transcription factor recipes used in reprogramming human cells into HSCs, related to Figure 2.**. Left: Recipes named by group and divided by the starting cell type. Right: TFs used in each recipe. *FH4a (and FH6) describe the 5TF recipe used throughout this manuscript.

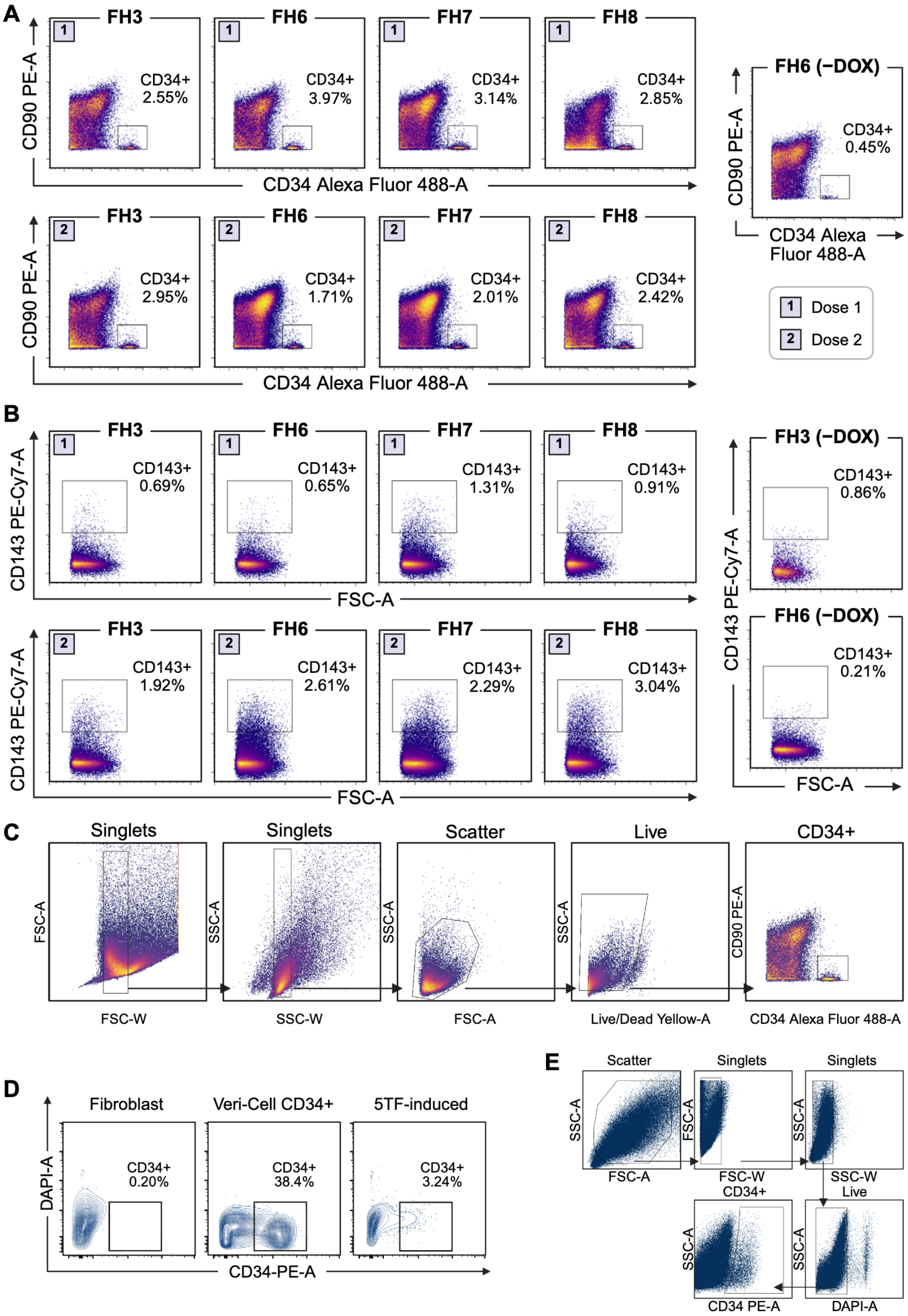

Figure S10: **Flow cytometry analysis of multiple TF recipes, related to Figure 2.** Different combinations of transcription factors in the 5TF recipe were tested for their ability to generate CD34$^+$ (A) and CD143$^+$ (B) cells. **(A)** Identification of CD34$^+$ cells with TF recipes (Table S7) delivered at low and high concentrations, corresponding to Dose 1 and Dose 2, respectively. FH6-transduced fibroblasts without Dox treatment served as negative controls. FH7 (GATA2, GFI1B, FOS, STAT5A) and FH8 (GATA2, GFI1B, FOS, REL) were prepared according to Method as described for FH3/FH6. Recipe combinations were made by mixing equal volumes of the corresponding lentiviral preparations. High and low dose amounts were based on identically prepared control fluorescent vectors. **(B)** Identification of CD143$^+$ cells. Right: FH6- and FH3-transduced fibroblasts without Dox treatment served as negative controls. **(C)** Gating scheme for hematopoietic markers, with the final gate shown for CD34. **(D)** Flow cytometry plots generated during flow sorting of the reprogramming cultures used for scRNA-seq. Left: BJ fibroblast negative control. Middle: Veri-Cell CD34$^+$ PBMCs (Biolegend) as a positive/gating control. Right: reprogramming cultures 48 days after initial Dox treatment, showing CD34$^+$ iHSCs. **(E)** Gating scheme for cell sorting. Created in BioRender. Cwycyshyn, J. (2026) https://BioRender.com/s83dtkl.

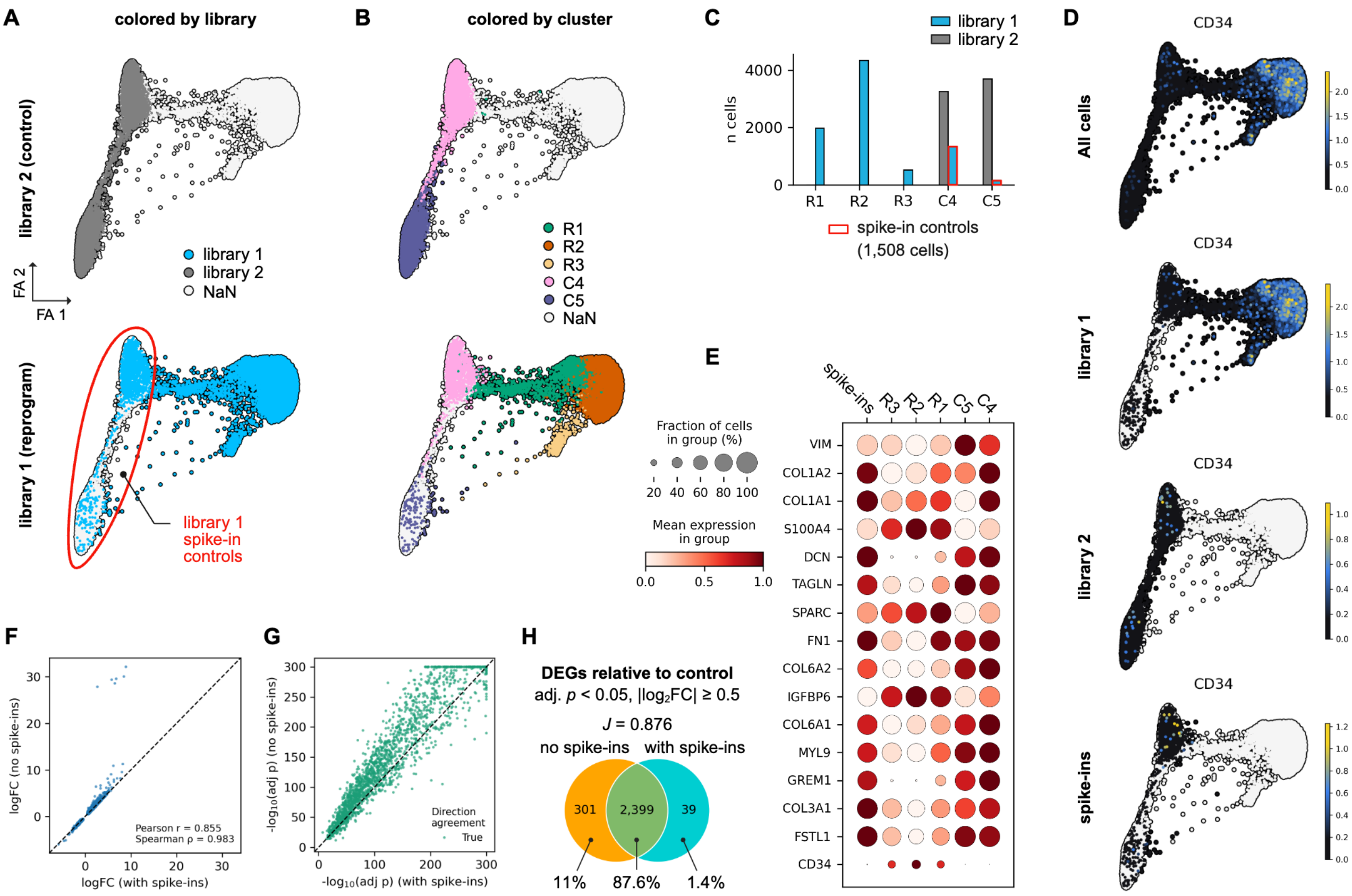

Figure S11: **Identification of spike-in CD34⁻ controls from library 1 (reprogram), related to Figure 2. (A)** Force-directed graph layout from Figure 2 colored by library and **(B)** by Leiden cluster for control (top) and reprogrammed (bottom) cells. **(C)** The number of cells in each cluster colored by library, with red boxes indicating the spike-in controls. **(D)** Log-normalized *CD34* expression on the graph from (A). Top to bottom: all cells from both libraries, only cells from library 1 (reprogram), only cells from library 2 (control), spike-in control cells (library 1 cells overlapping with control clusters). **(E)** Mean log-normalized expression of *CD34* and the top 15 fibroblast marker genes expressed in controls by cluster (spike-ins plotted separately). **(F)** Comparison of $\log_2$ fold change (logFC) values for significant DEGs between reprogrammed and control cells when spike-in controls were excluded versus included in the control population (Wilcoxon Rank-sum test, corrected $\alpha = 0.05$, $|\log_2$ fold change$| > 0.5$, expressed in $> 30\%$ of cells where $\log_2$ fold change > 0). Each point represents a shared DEG; the dashed line denotes identity. Pearson (r) and Spearman (ρ) correlation coefficients are shown. **(G)** Comparison of statistical significance (-$\log_{10}$(adjusted p-value)) for DEGs from (F). Points are colored by agreement in direction of differential expression between analyses; the dashed line denotes identity. **(H)** Overlap of DEGs from (F). Percentages indicate the fraction of total unique DEGs across both analyses; Jaccard index ($J = 0.876$). Created in BioRender. Cwycyshyn, J. (2026) https://BioRender.com/b7tq437.

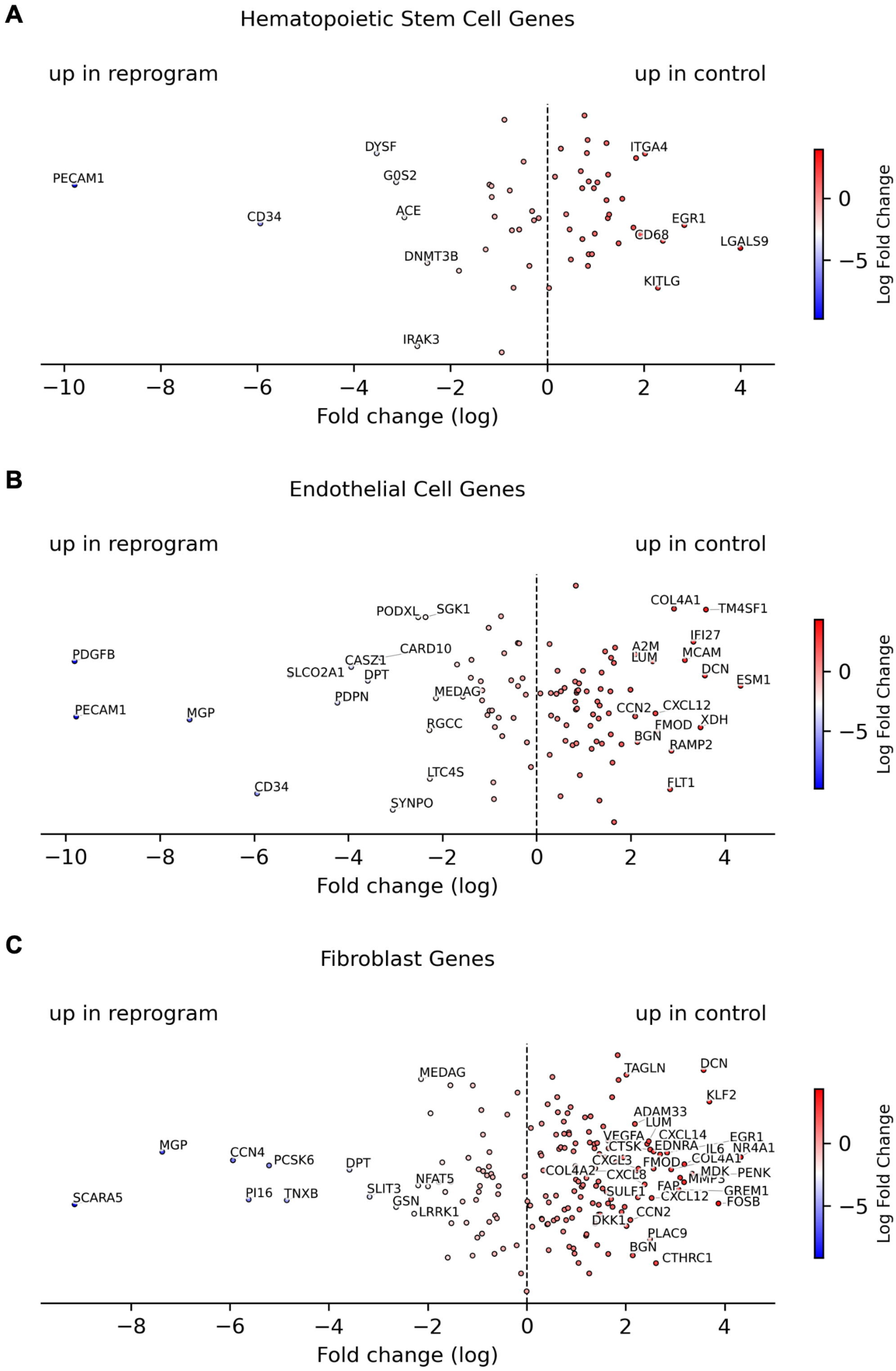

Figure S12: **Differentially expressed marker gene sets, related to Figure 2.** Differential expression results for marker gene sets from PanglaoDB[2] between reprogrammed cells and controls (Wilcoxon rank sum, corrected $\alpha = 0.05$). All plots highlight genes with absolute $\log_2$ fold change > 2. **(A)** HSC genes. **(B)** Endothelial genes. **(C)** Fibroblast genes. Created in BioRender. Cwycyshyn, J. (2026) https://BioRender.com/g1hgprg.

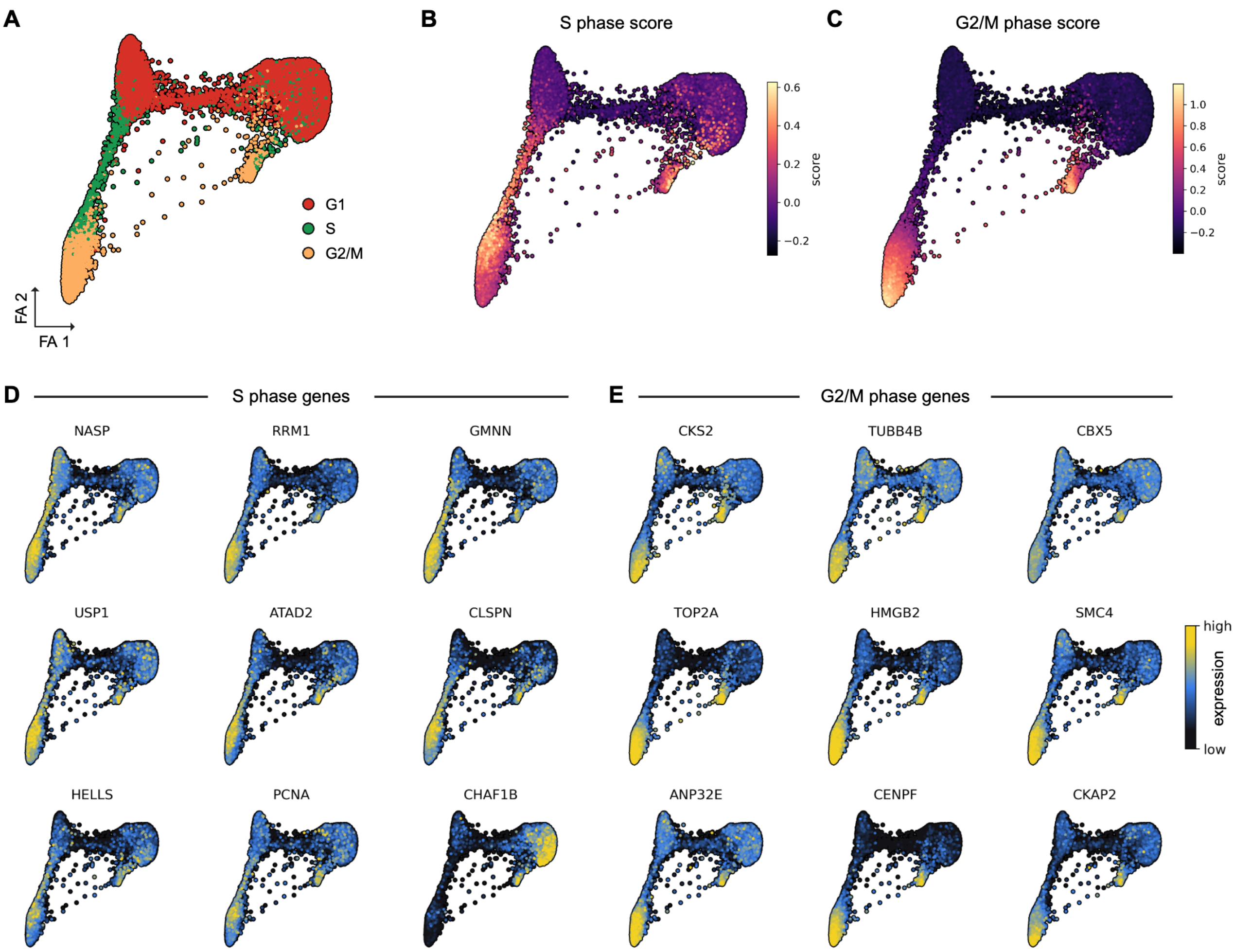

Figure S13: **Cell cycle phase analysis for control and reprogrammed cells, related to Figure 3.** **(A)** Predicted cell cycle phase for all cells. **(B)** S-phase scores and **(C)** G2/M-phase scores. Scores were computed using the `scanpy.tl.score_genes_cell_cycle` function with gene sets from[3]. **(D)** Log-normalized expression of top expressed S-phase genes. **(E)** Log-normalized expression of top expressed G2/M-phase genes. Created in BioRender. Cwycyshyn, J. (2026) https://BioRender.com/eicidwt.

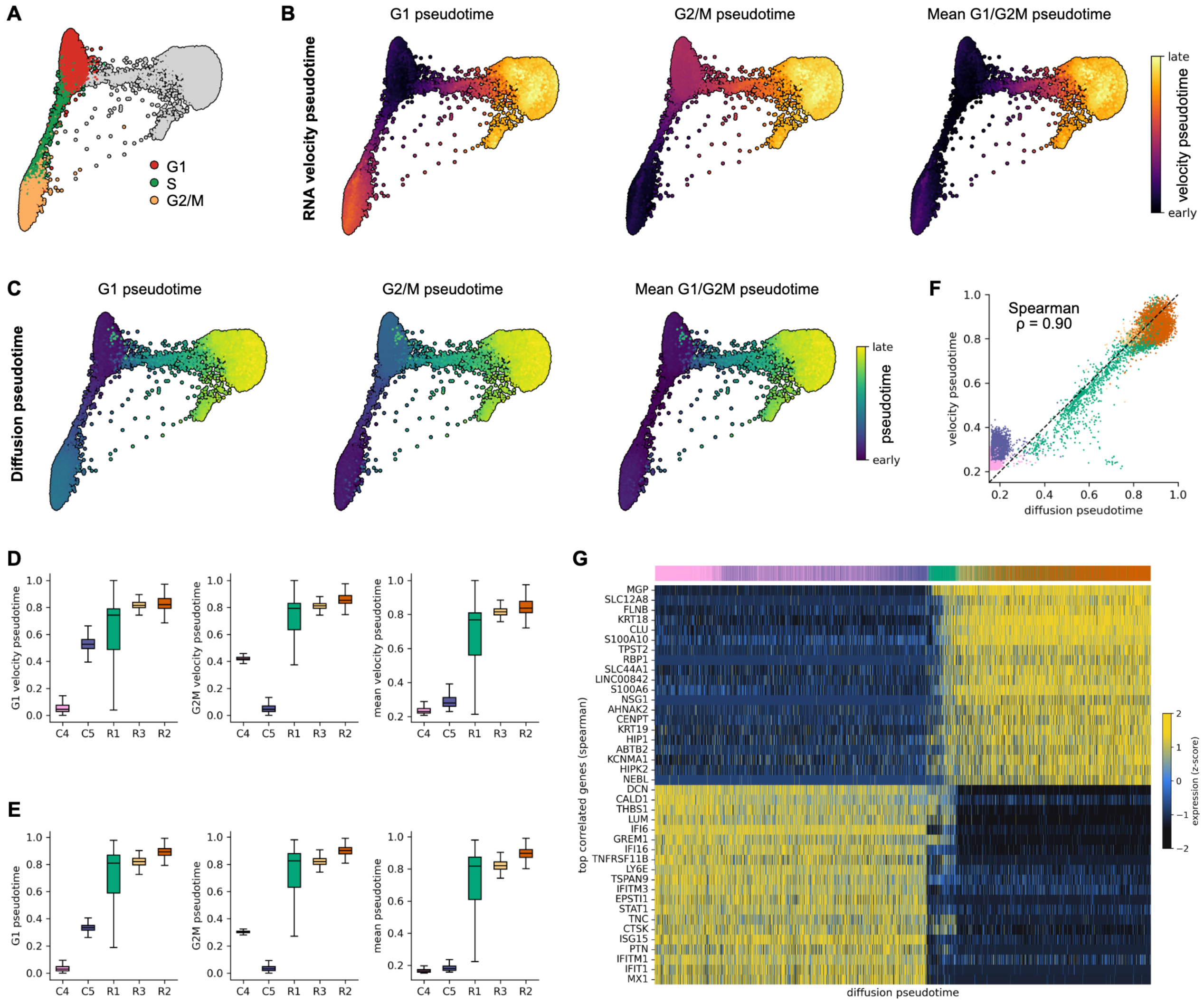

Figure S14: **Comparison between diffusion-based pseudotime and RNA velocity pseudotime, related to Figure 3. (A)** Predicted cell cycle phase for cells from Library 2 (control) used for selecting root cells in pseudotemporal orderings. **(B)** Cells colored by RNA velocity pseudotime with root cells in G1, G2/M, and averaged G1 and G2/M (left to right). **(C)** Cells colored by diffusion-based pseudotime with root cells in G1, G2/M, and averaged G1 and G2/M (left to right). **(D)** Per cluster distributions of velocity-based pseudotemporal orderings and **(E)** diffusion-based pseudotemporal orderings (Data are represented as median and IQR, with whiskers extending to $1.5 \times$ IQR). **(F)** Correlation between RNA velocity pseudotime and diffusion-based pseudotime (Spearman) colored by cell cluster (Spearman $\rho = 0.90$). Dashed line indicates identity. **(G)** Z-scored expression of top positively and negatively correlated genes with diffusion-based pseudotime. Cells (columns) are ordered by diffusion pseudotime and annotated by cluster (color bar, top). Correlations were computed using Spearman's ρ, and significance was assessed by Benjamini-Hochberg FDR correction ($\alpha = 0.05$). Only genes with adjusted $p < 0.05$ were considered. Created in BioRender. Cwycyshyn, J. (2026) https://BioRender.com/4g6cgx7.

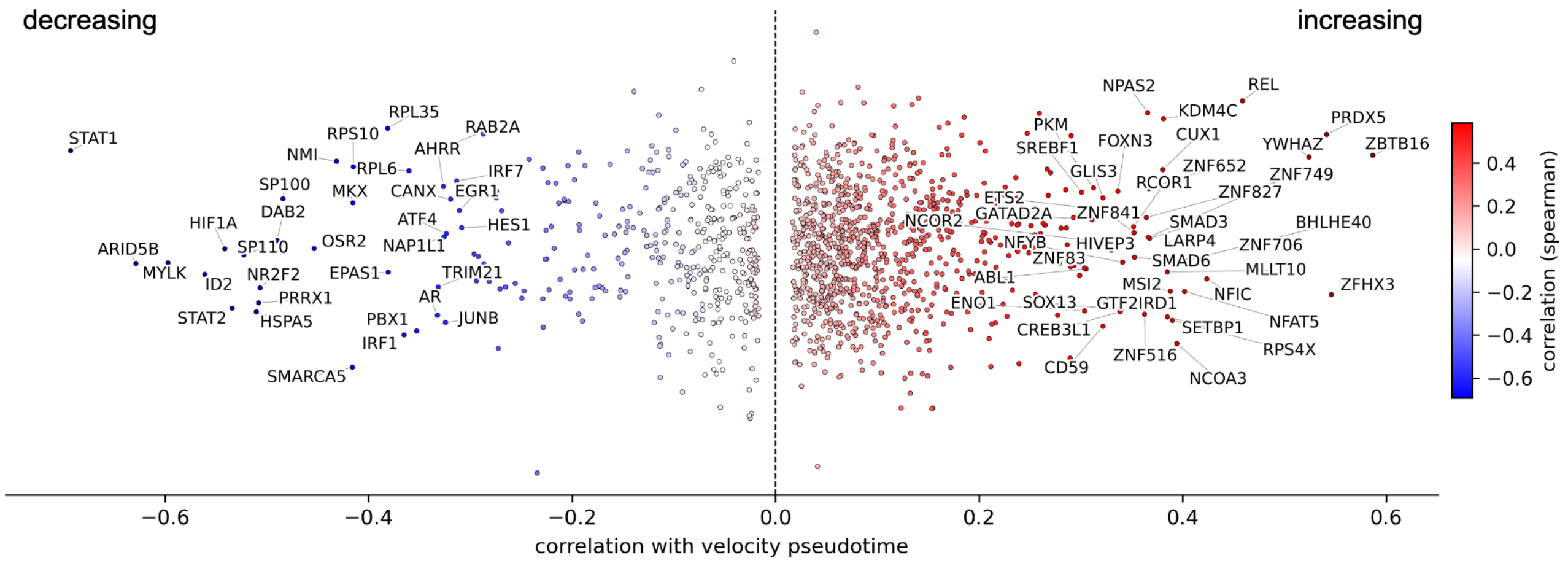

Figure S15: **Correlation of transcription factor expression with RNA velocity pseudotime, related to Figure 3.** Each point represents a TF, colored by its Spearman correlation with velocity pseudotime. Only TFs with Benjamini-Hochberg FDR-adjusted $p < 0.05$ are shown. Jitter is added along the y-axis for visual clarity. TFs with $|\rho| > 0.3$ are labeled. Created in BioRender. Cwycyshyn, J. (2026) https://BioRender.com/74lqdwl.

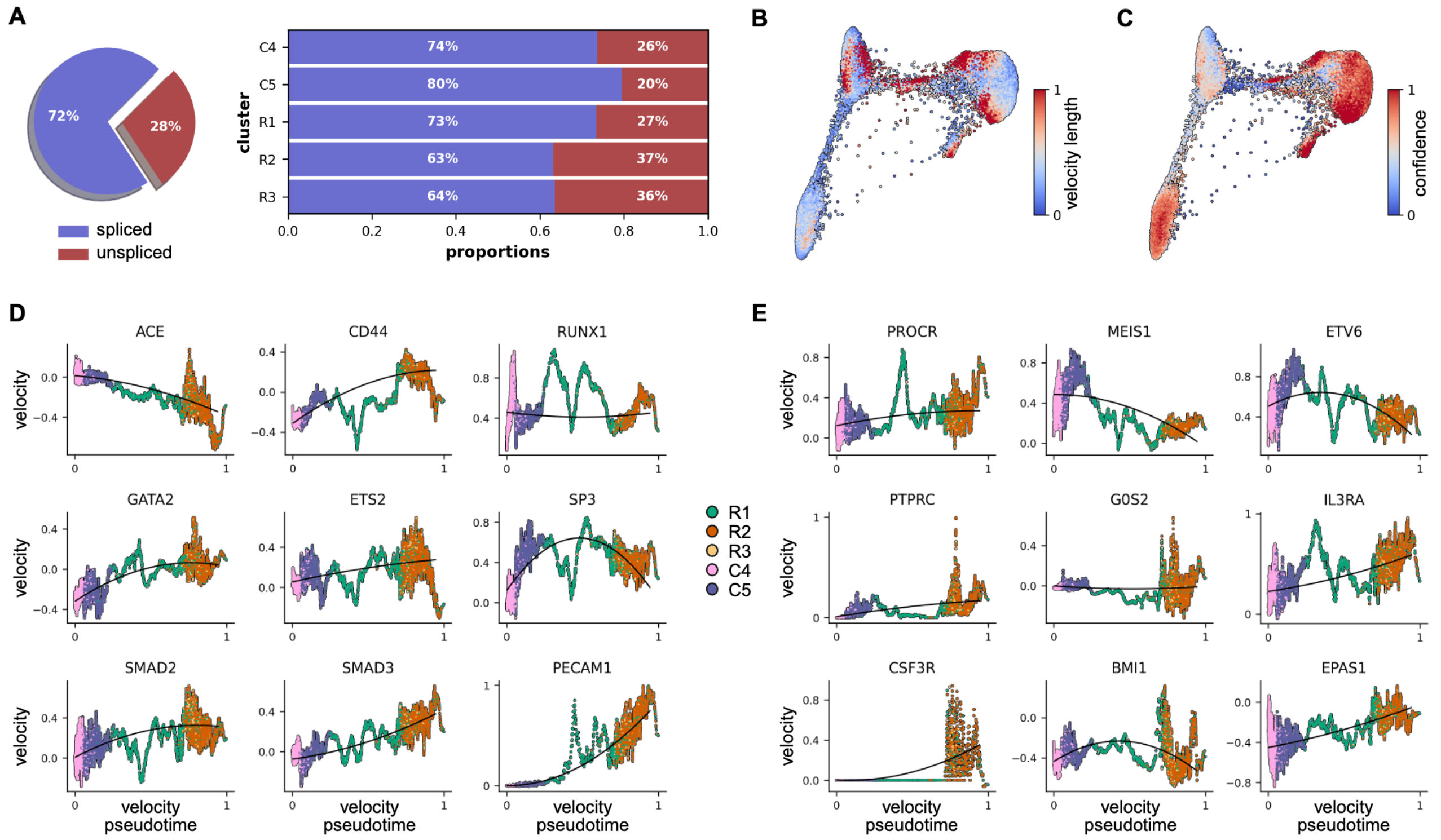

Figure S16: **Extended RNA velocity analysis, related to Figure 3. (A)** Spliced and unspliced transcript proportions by cluster. **(B)** Length of velocity vectors indicative of the rate of differentiation. **(C)** Confidence of velocities indicative of where vector direction is un-/determined. **(D)** Velocity shown over velocity pseudotime for endothelial/EHT-related genes and **(E)** HSC genes colored by Leiden cluster. Velocity values represent the inferred rate of change of gene expression for each gene in each cell, with positive values indicating increasing expression. Created in BioRender. Cwycyshyn, J. (2026) https://BioRender.com/lw3t29o.

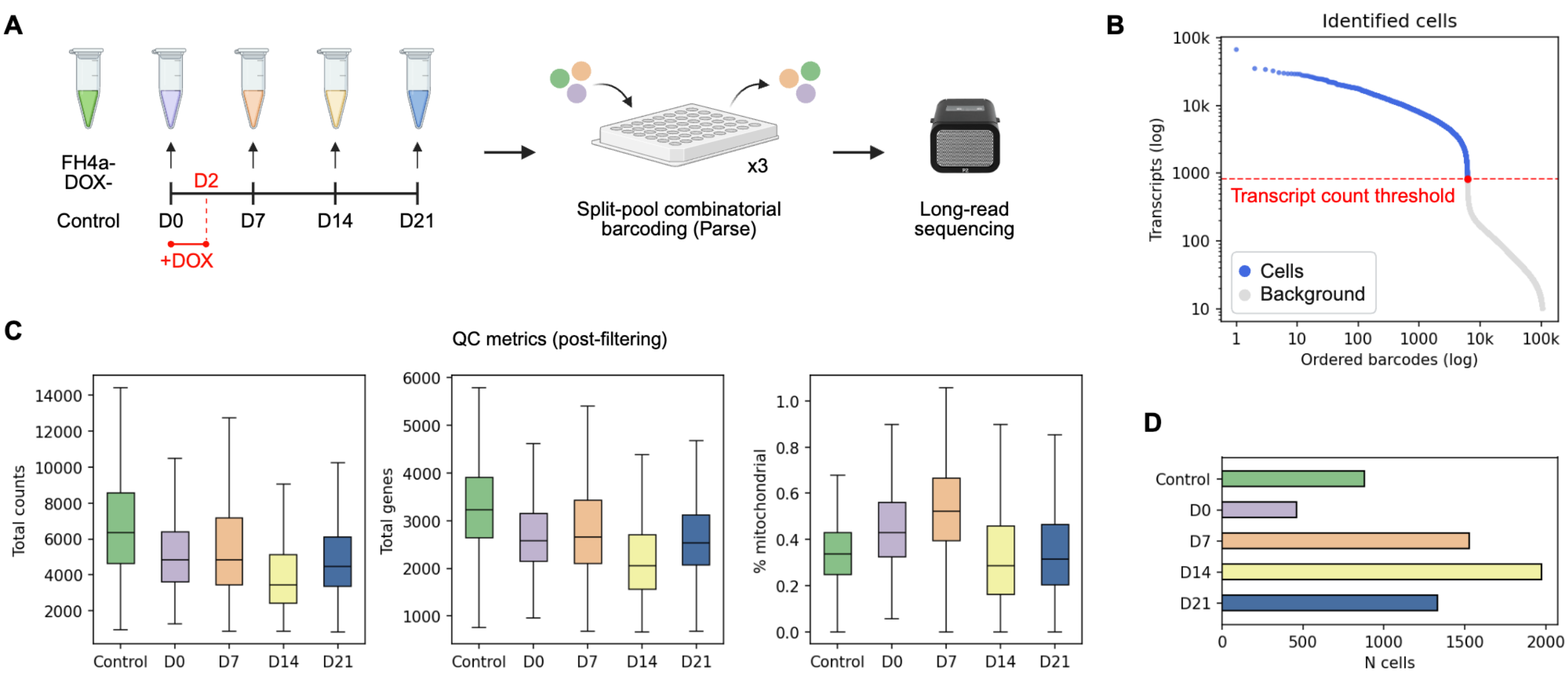

Figure S17: **Time-series reprogramming dataset collection and quality control, related to Figure 3. (A)** 5TF cells were collected on days 0, 7, 14, and 21 of reprogramming. A sample of non-transduced Dox- fibroblasts served as an additional control. Single cells were barcoded via split-pool combinatorial barcoding and sequencing on the ONT platform. **(B)** Barcode rank plot showing the transcript count threshold used to distinguish cells from background. **(C)** Quality control metrics after removal of low quality cells for each time point (Data are represented as median and IQR, with whiskers extending to $1.5\times$ IQR) **(D)** The total number of cells per time point post-filtering. Created in BioRender. Cwycyshyn, J. (2026) https://BioRender.com/7gg8zb1.

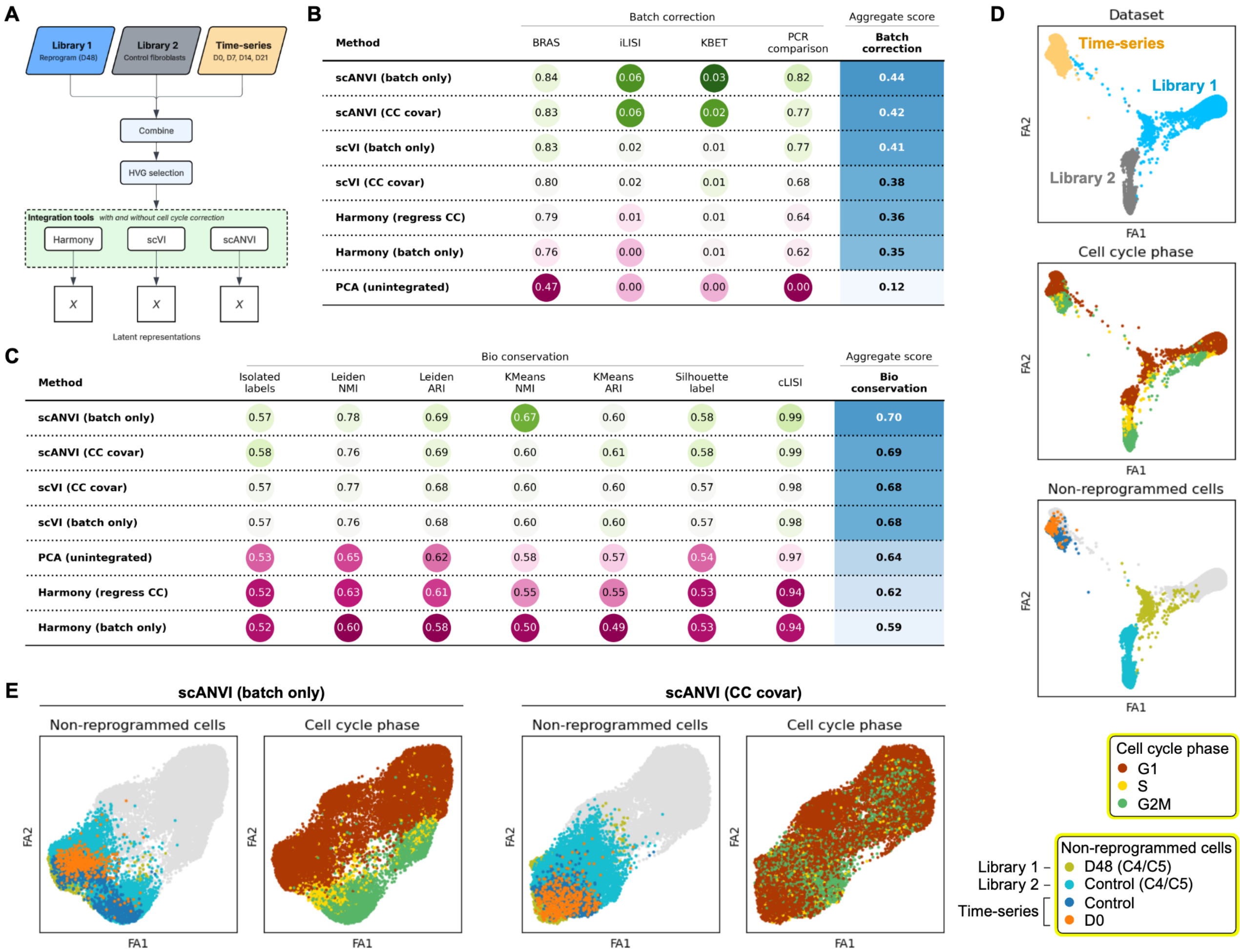

	Batch correction				Aggregate score
Method	BRAS	iLISI	KBET	PCR comparison	Batch correction
scANVI (batch only)	0.84	0.06	0.03	0.82	0.44
scANVI (CC covar)	0.83	0.06	0.02	0.77	0.42
scVI (batch only)	0.83	0.02	0.01	0.77	0.41
scVI (CC covar)	0.80	0.02	0.01	0.68	0.38
Harmony (regress CC)	0.79	0.01	0.01	0.64	0.36
Harmony (batch only)	0.76	0.00	0.01	0.62	0.35
PCA (unintegrated)	0.47	0.00	0.00	0.00	0.12

	Bio conservation							Aggregate score
Method	Isolated labels	Leiden NMI	Leiden ARI	KMeans NMI	KMeans ARI	Silhouette label	cLISI	Bio conservation
scANVI (batch only)	0.57	0.78	0.69	0.67	0.60	0.58	0.99	0.70
scANVI (CC covar)	0.58	0.76	0.69	0.60	0.61	0.58	0.99	0.69
scVI (CC covar)	0.57	0.77	0.68	0.60	0.60	0.57	0.98	0.68
scVI (batch only)	0.57	0.76	0.68	0.60	0.60	0.57	0.98	0.68
PCA (unintegrated)	0.53	0.65	0.62	0.58	0.57	0.54	0.97	0.64
Harmony (regress CC)	0.52	0.63	0.61	0.55	0.55	0.53	0.94	0.62
Harmony (batch only)	0.52	0.60	0.58	0.50	0.49	0.53	0.94	0.59

Figure S18: **Time-series dataset integration and benchmarking, related to STAR Methods. (A)** Schematic of the integration process. Shared low-dimensional representations were generated using three integration tools (Harmony[4], scVI[5], scANVI[6]) with and without cell cycle correction. **(B)** Batch correction benchmarking results performed on non-reprogrammed cells from each of the three datasets. **(C)** Biological conservation benchmarking results performed on cells from the time-series dataset. **(D)** Force-directed graph layouts generated from the unintegrated PCA representation, colored by dataset, cell cycle phase, and non-reprogrammed cells (top to bottom). **(E)** Force-directed graph layouts generated from the scANVI representation colored by non-reprogrammed cells and cell cycle phase. Left: Batch (dataset) was the only covariate; Right: Cell cycle phase scores were continuous covariates in addition to batch. Created in BioRender. Cwycyshyn, J. (2026) https://BioRender.com/kyferdp.

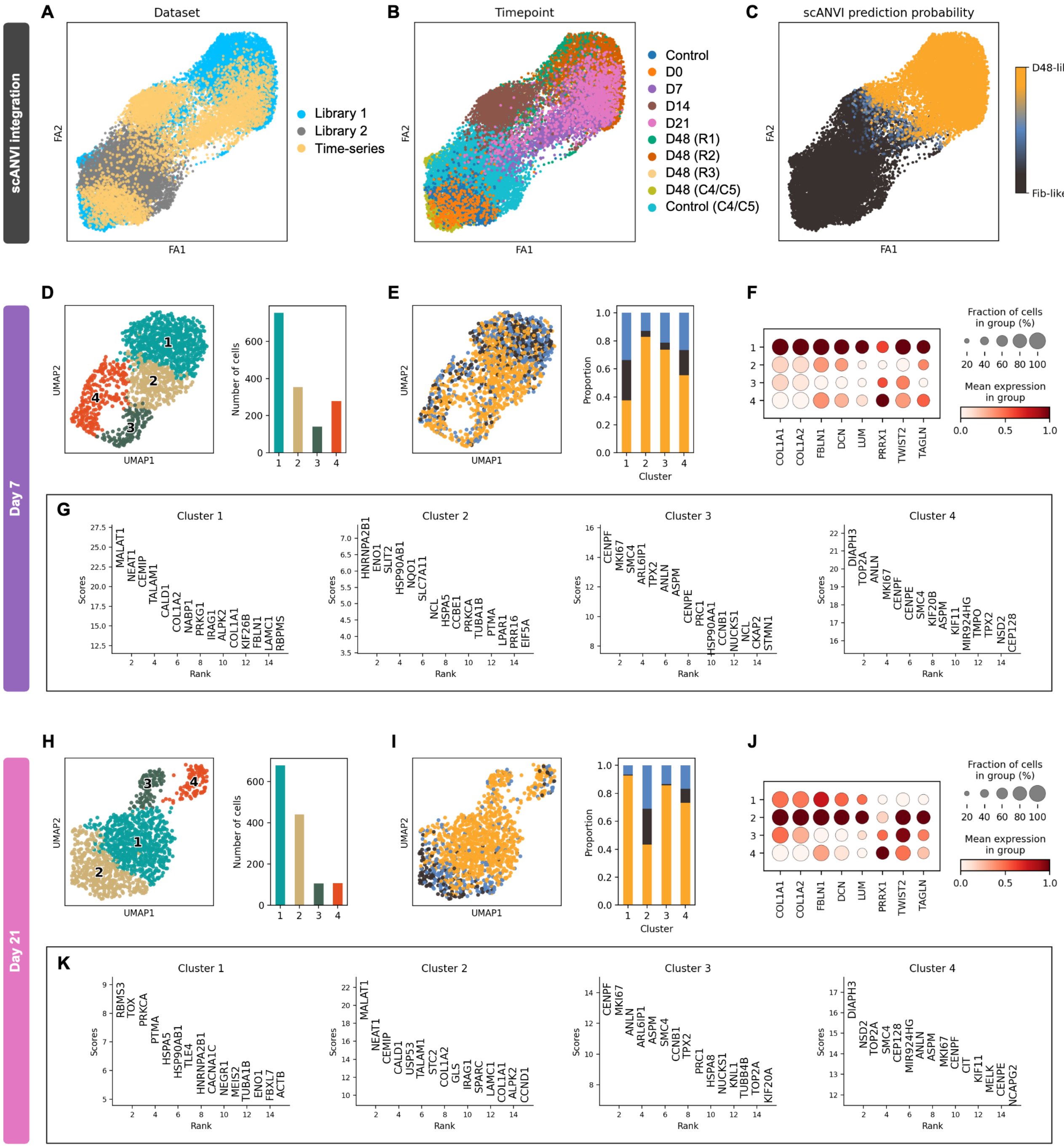

Figure S19: **scANVI predictions and clustering of time-series cells, related to Figure 3. (A)** Force-directed graph layout colored by dataset generated from the scANVI latent representation with cell cycle covariates. **(B)** Graph from (A) colored by timepoint. **(C)** Graph from (A) colored by scANVI prediction probability for fibroblast-like or day 48-like cell classes. **(D)** Left: UMAP plot of day 7 cells colored by Leiden cluster; Right: Number of cells per cluster. **(E)** Left: Day 7 UMAP colored by scANVI-predicted cell class; Right: Distribution of predicted cell classes across clusters. **(F)** Log-normalized expression (scaled from $0-1$) for select fibroblast marker genes across day 7 clusters. **(G)** Top 15 DEGs for each day 7 cluster ranked by z-score (Wilcoxon Rank-sum test, corrected $\alpha = 0.05$). **(H)** Left: UMAP plot of day 21 cells colored by Leiden cluster; Right: Number of cells per cluster. **(I)** Left: Day 21 UMAP colored by scANVI-predicted cell class; Right: Distribution of predicted cell classes across clusters. **(J)** Log-normalized expression (scaled from $0-1$) for select fibroblast marker genes across day 21 clusters. **(K)** Top 15 DEGs for each day 21 cluster ranked by z-score (Wilcoxon Rank-sum test, corrected $\alpha = 0.05$). Created in BioRender. Cwycyshyn, J. (2026) https://BioRender.com/rwgxf09.

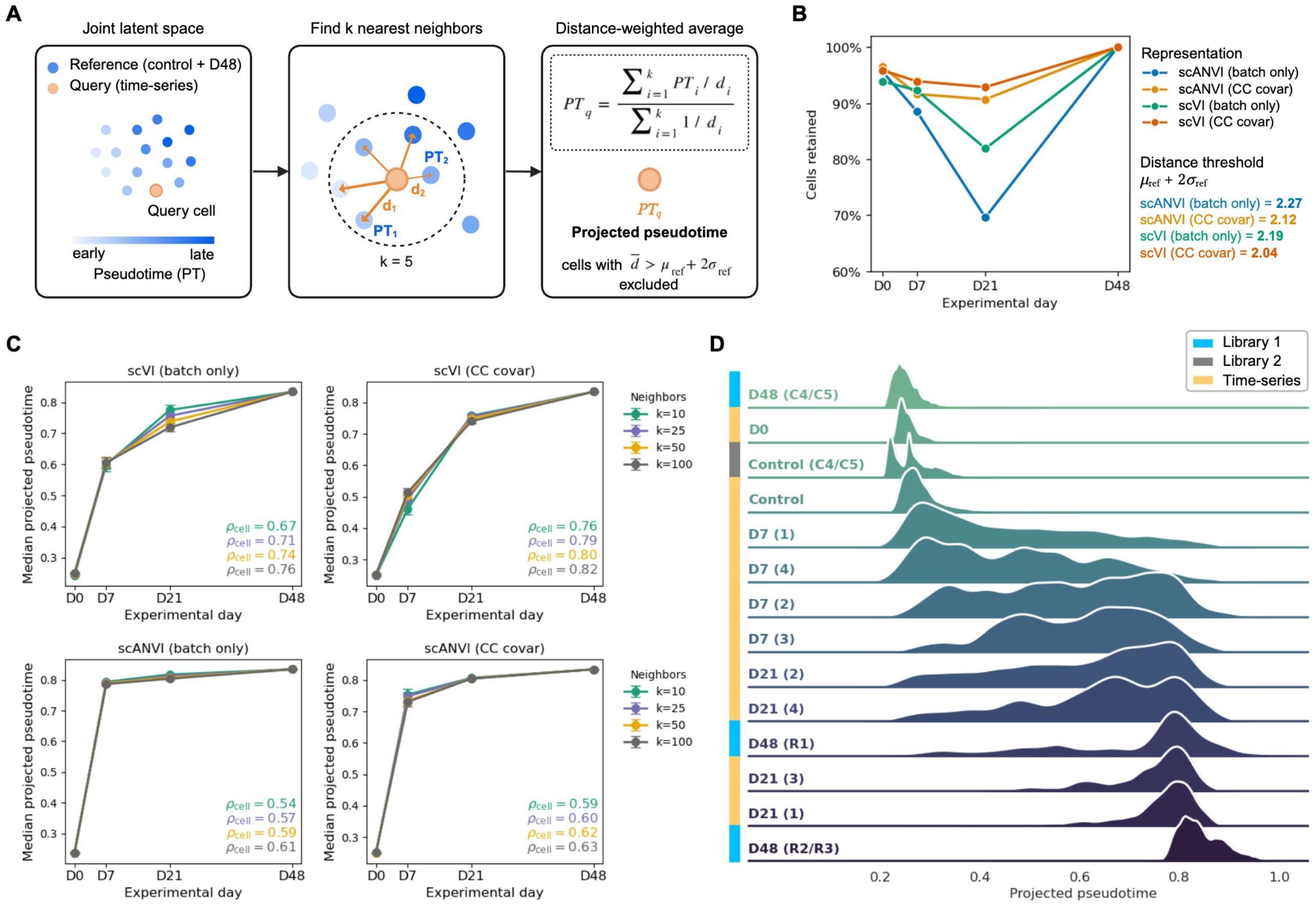

Figure S20: **Pseudotime projection, related to Figure 3. (A)** Schematic of the pseudotime projection method. **(B)** The percentage of cells retained per timepoint after dropping low coverage cells. Lines are colored by latent representation. Query cells (time-series cells) with a mean neighbor distance less than the indicated thresholds were retained. **(C)** Median projected pseudotime values per timepoint for each latent representation. Error bars represent bootstrapped 95% CIs. Lines are colored by the number of nearest neighbors used in the projection. Spearman's correlation (ρ_{cell}) was computed on per-cell distributions of projected pseudotime values versus experimental day. **(D)** Density plots of projected pseudotime distributions per cluster-resolved timepoint. The scVI representation with cell cycle covariates was used for projection with $k = 25$ nearest neighbors. Clusters for Libraries 1 and 2 are derived from Fig. 2F. Clusters for time-series cells are derived from Supplementary Fig. S19D and H. Spearman's ρ values were computed on per-cell distributions of projected pseudotime values (with scVI and scANVI representations) versus experimental day across the clusters indicated by the red dots. Created in BioRender. Cwycyshyn, J. (2026) https://BioRender.com/kc5ubcu.

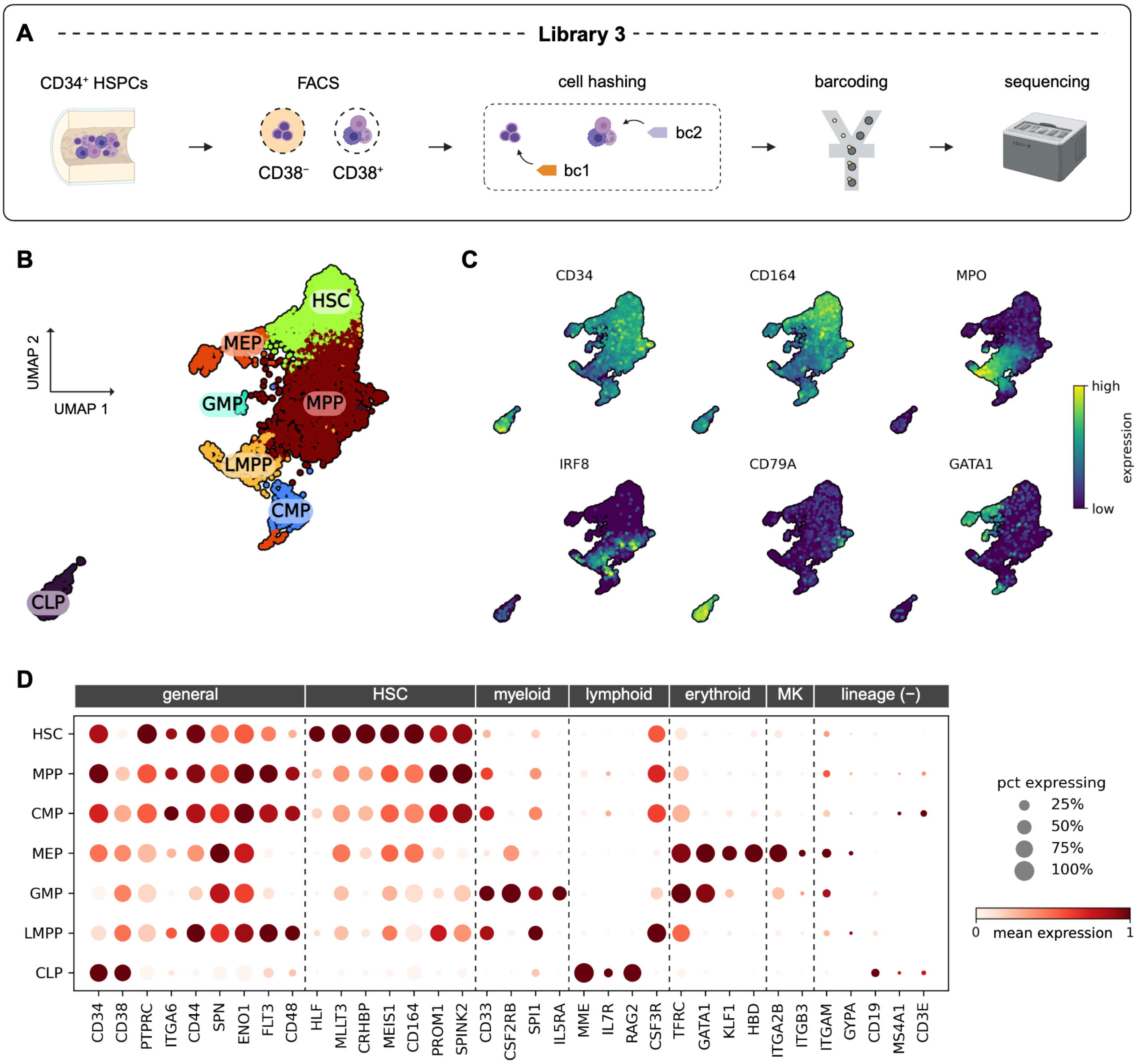

Figure S21: **Characterization of CD34$^+$ human bone marrow scRNA-seq, related to Figure 4. (A)** Experimental workflow for scRNA-seq of human bone marrow cells (Library 3), including cell hashing of FACS-sorted CD38$^+$ and CD38$^-$ populations. **(B)** UMAP of cells colored by Leiden cluster and labeled by cell type annotations. MPP: multipotent progenitor, CMP: common myeloid progenitor, CLP: common lymphoid progenitor, LMPP: lymphoid-primed multipotent progenitor, GMP: granulocyte-monocyte progenitor, MEP: megakaryocyte-erythroid progenitor. **(C)** Normalized expression of key hematopoietic transcripts[7] on UMAP coordinates from (B). **(D)** Dot plot showing mean log-normalized expression of lineage-specific marker genes per cell type. Dot size represents the percentage of cells expressing a given gene. Created in BioRender. Cwycyshyn, J. (2026) https://BioRender.com/ij8lvs5.

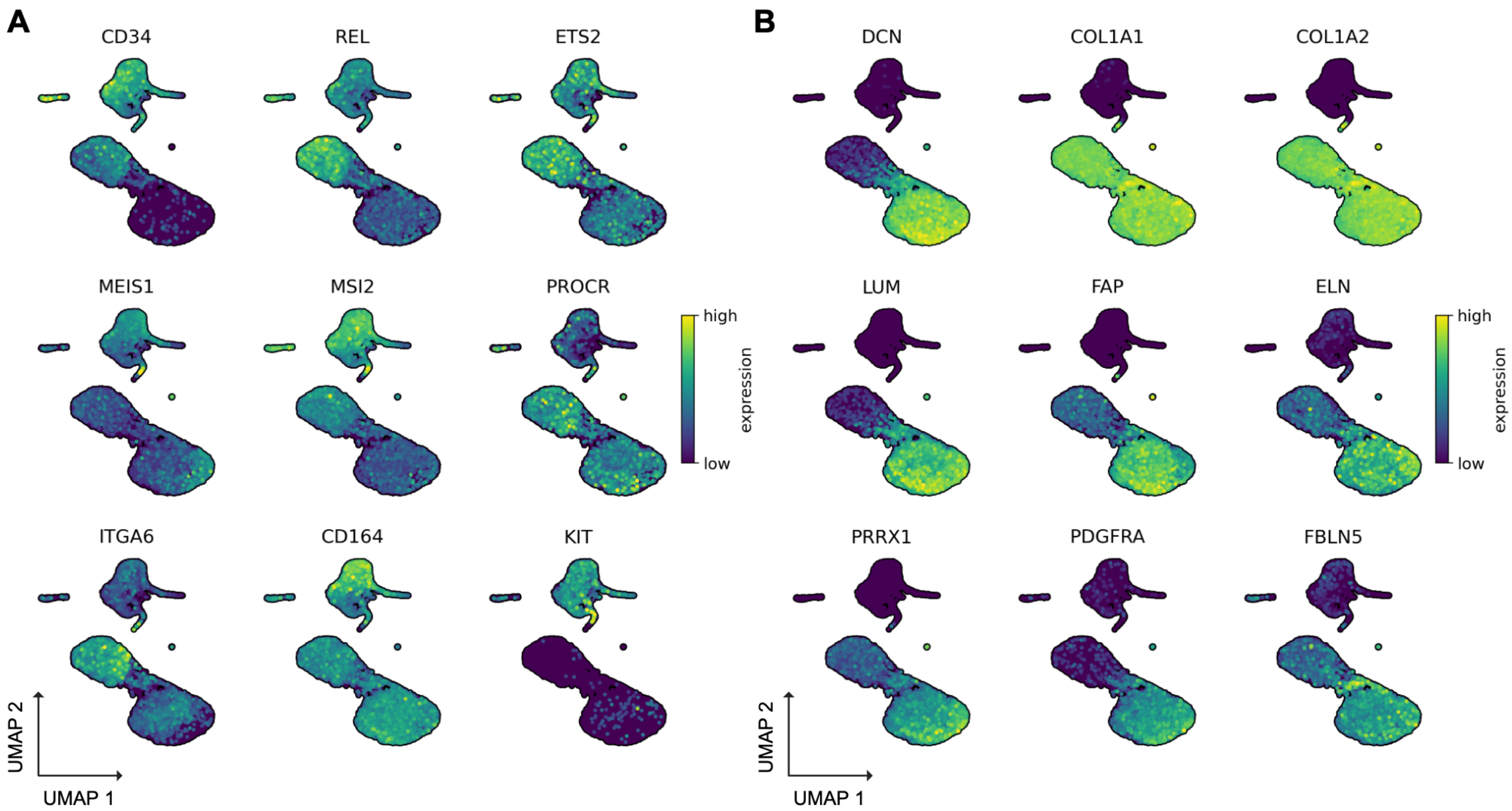

Figure S22: **Marker gene expression in initial, reprogrammed, and target cells, related to Figure 4. (A)** Log-normalized expression of hematopoietic genes and **(B)** fibroblast genes on the UMAP coordinates from Figure 4B. Created in BioRender. Cwycyshyn, J. (2026) https://BioRender.com/nn98ak3.

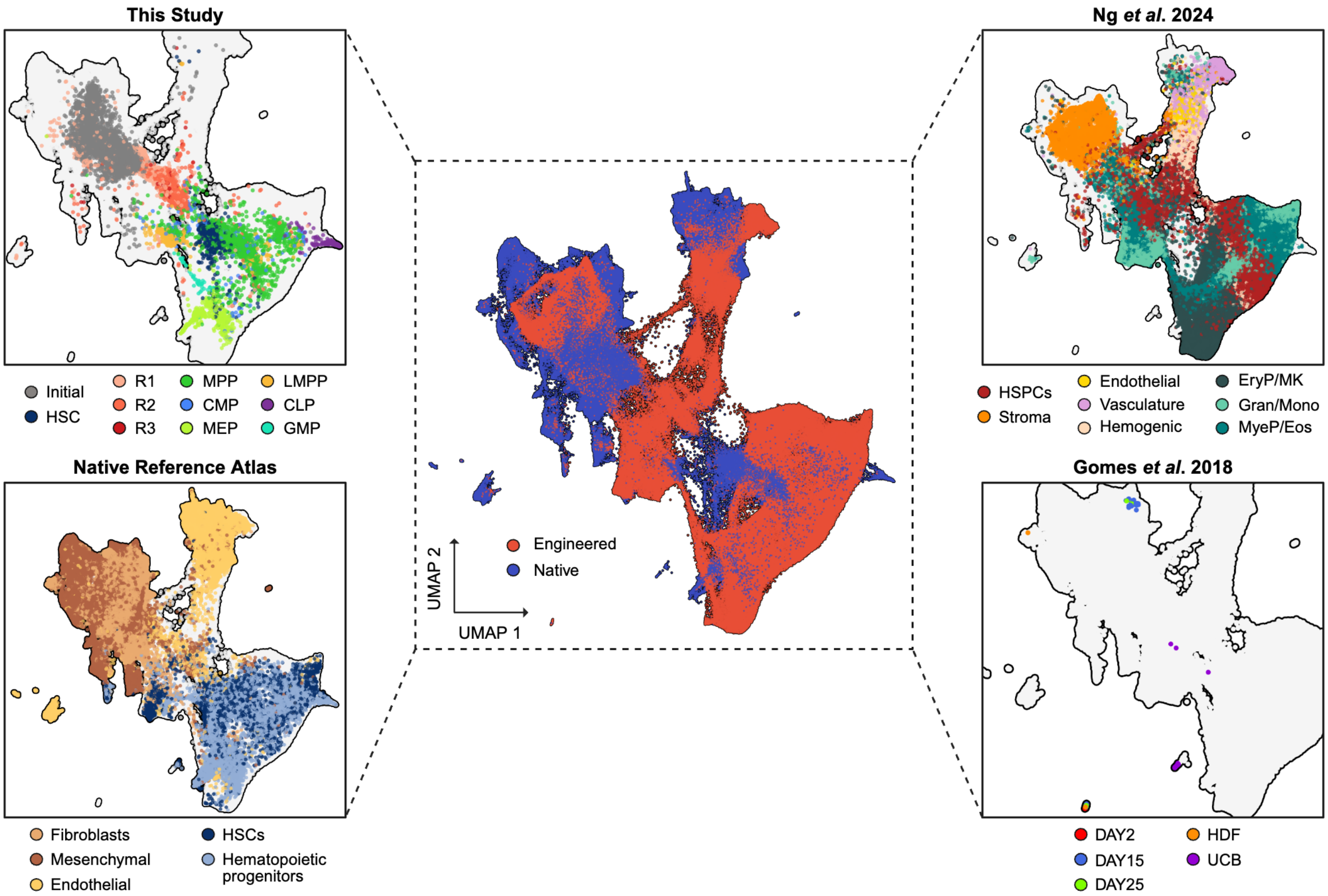

Figure S23: **Overlap between data sources in the integrated atlas, related to Figure 4. Center:** UMAP of 420,108 cells from the native reference atlas, this study, and selected sources based on scVI latent space, colored by engineered or native cell types. **Top left:** Same UMAP showing the 20,620 cells from this study (reprogrammed, control, CD34+ human bone marrow) colored by their cell type annotations. HSC: hematopoietic stem cell, MPP: multipotent progenitor, CMP: common myeloid progenitor, MEP: megakaryocyte-erythroid progenitor, LMPP: lymphoid-primed multipotent progenitor, CLP: common lymphoid progenitor, GMP: granulocyte-monocyte progenitor; **Bottom left:** Same UMAP showing 167,133 cells from the native reference atlas colored by cell annotation; **Top right:** Same UMAP showing 232,072 engineered cells from[8]. These cells underwent in vitro differentiation into the hematopoietic lineage from induced pluripotent stem cells, and annotations from the source were consolidated into broader annotations. HSPCs: hematopoietic stem and progenitor cells, EryP/MK: erythroid-megakaryocyte progenitor/megakaryocyte, Gran/Mono: granulocyte/monocyte, MyeP/Eos: myeloid progenitor/eosinophil; **Bottom right:** Same UMAP showing 283 control and reprogrammed cells from[9]. Reprogrammed cells were induced with the 3TF recipe (GATA2, GFI1B, FOS). HDF: human dermal fibroblasts, UCB: umbilical cord blood (Lin-CD34+), DAY2: 3TF-induced day 2, DAY15: 3TF-induced CD49f+ day 15, DAY25: 3TF-induced CD49f+ day 25. Created in BioRender. Cwycyshyn, J. (2026) https://BioRender.com/78qpbm8.

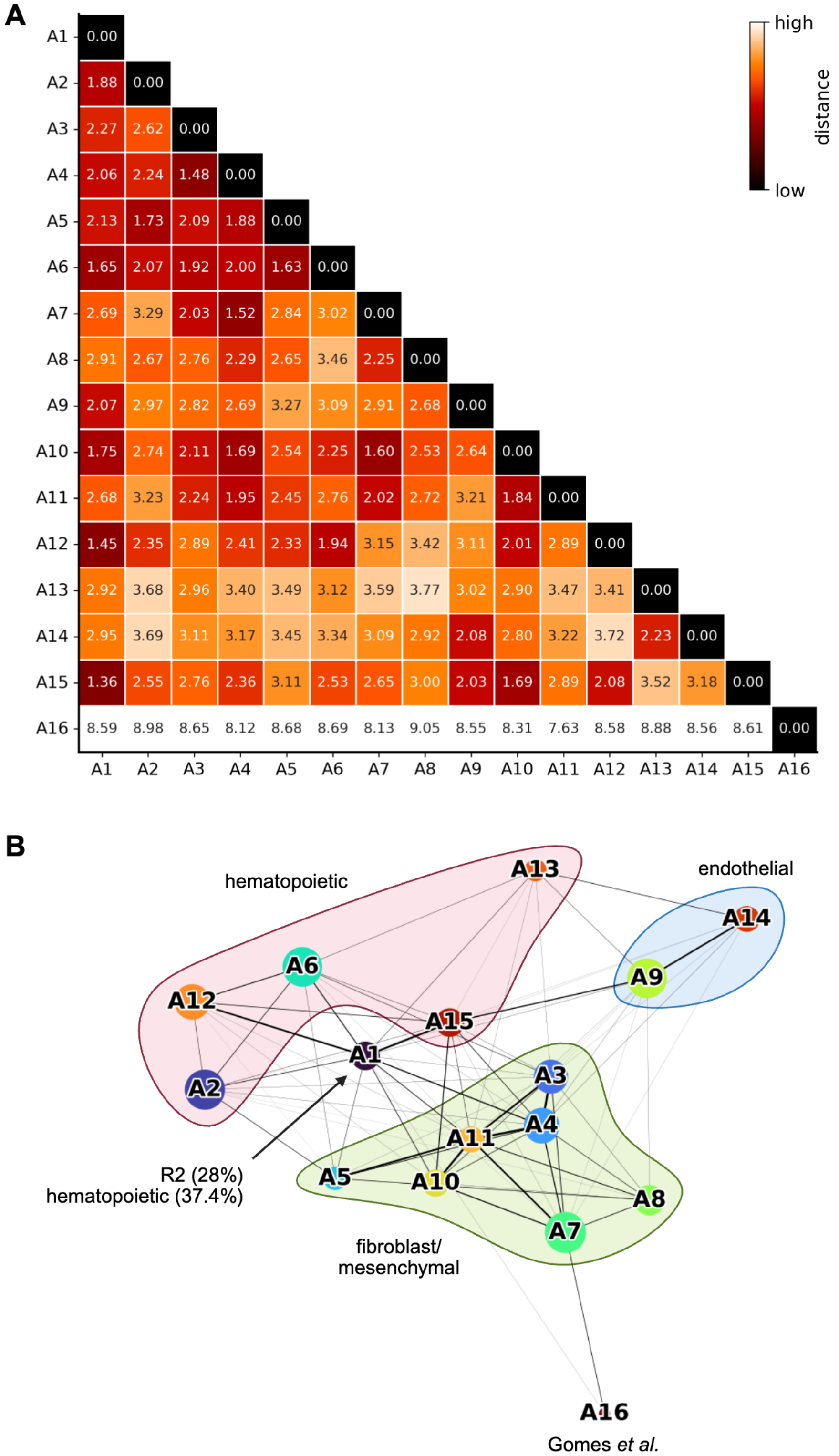

Figure S24: **Pairwise Euclidean distances between clusters in the integrated atlas, related to Figure 4. (A)** Euclidean distance between mean scVI latent vectors for clusters in the integrated atlas dataset. **(B)** PAGA plot[10] of clusters in the integrated atlas with broad annotations based on most common cell type composition (Table S5). Created in BioRender. Cwycyshyn, J. (2026) https://BioRender.com/4raengu.

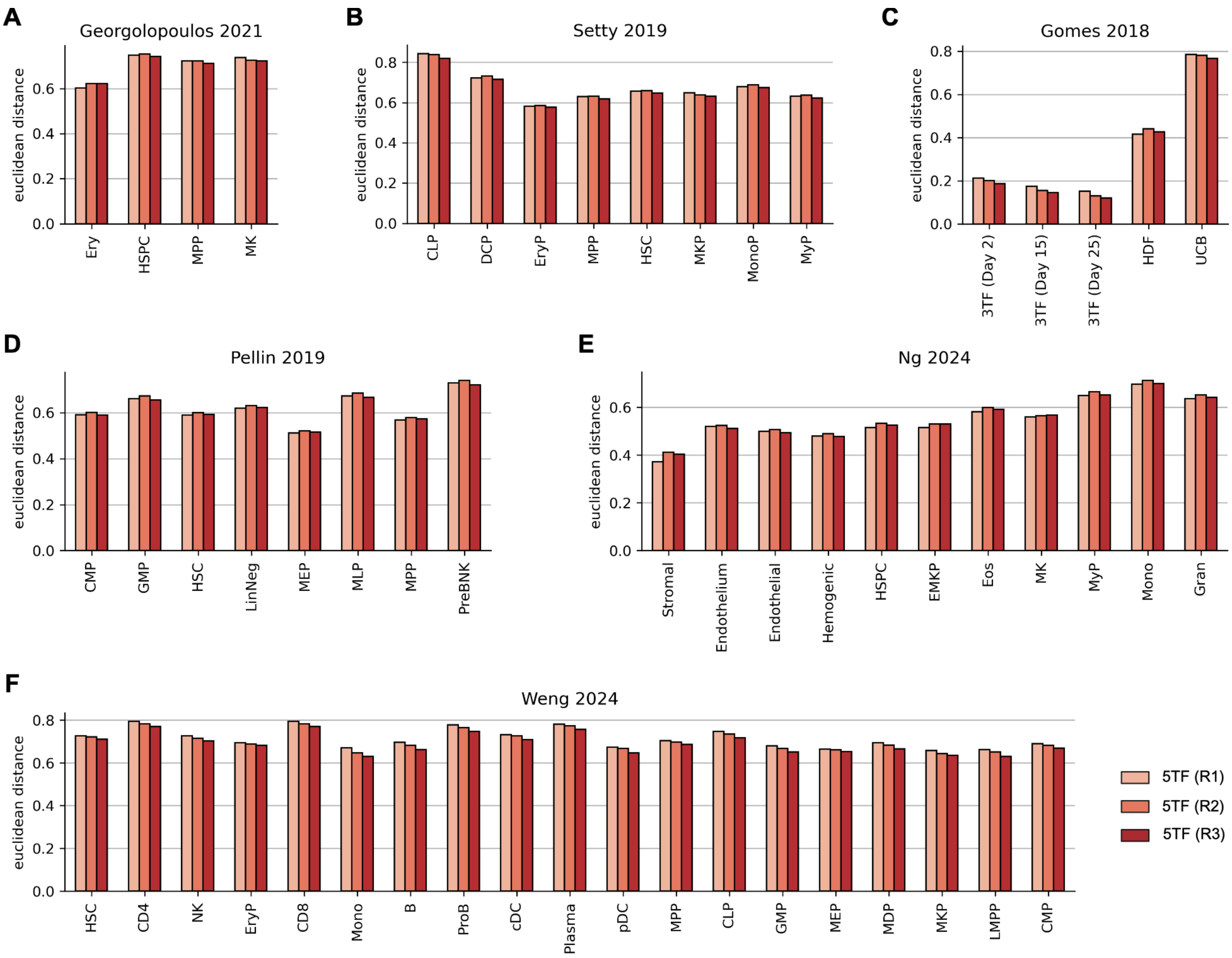

Figure S25: **Pseudobulk distances between reprogrammed cells and datasets from literature, related to Figure 4. (A)** Distances to cell types in[11]. **(B)** Distances to cell types in[7]. **(C)** Distances to cell types in[9]. **(D)** Distances to cell types in[12]. **(E)** Distances to cell types in[8]. **(F)** Distances to cell types in[13]. Bars indicate distances (min-max normalized) computed independently for each reprogramming cluster (R1–R3). Created in BioRender. Cwycyshyn, J. (2026) https://BioRender.com/9xlgegc.

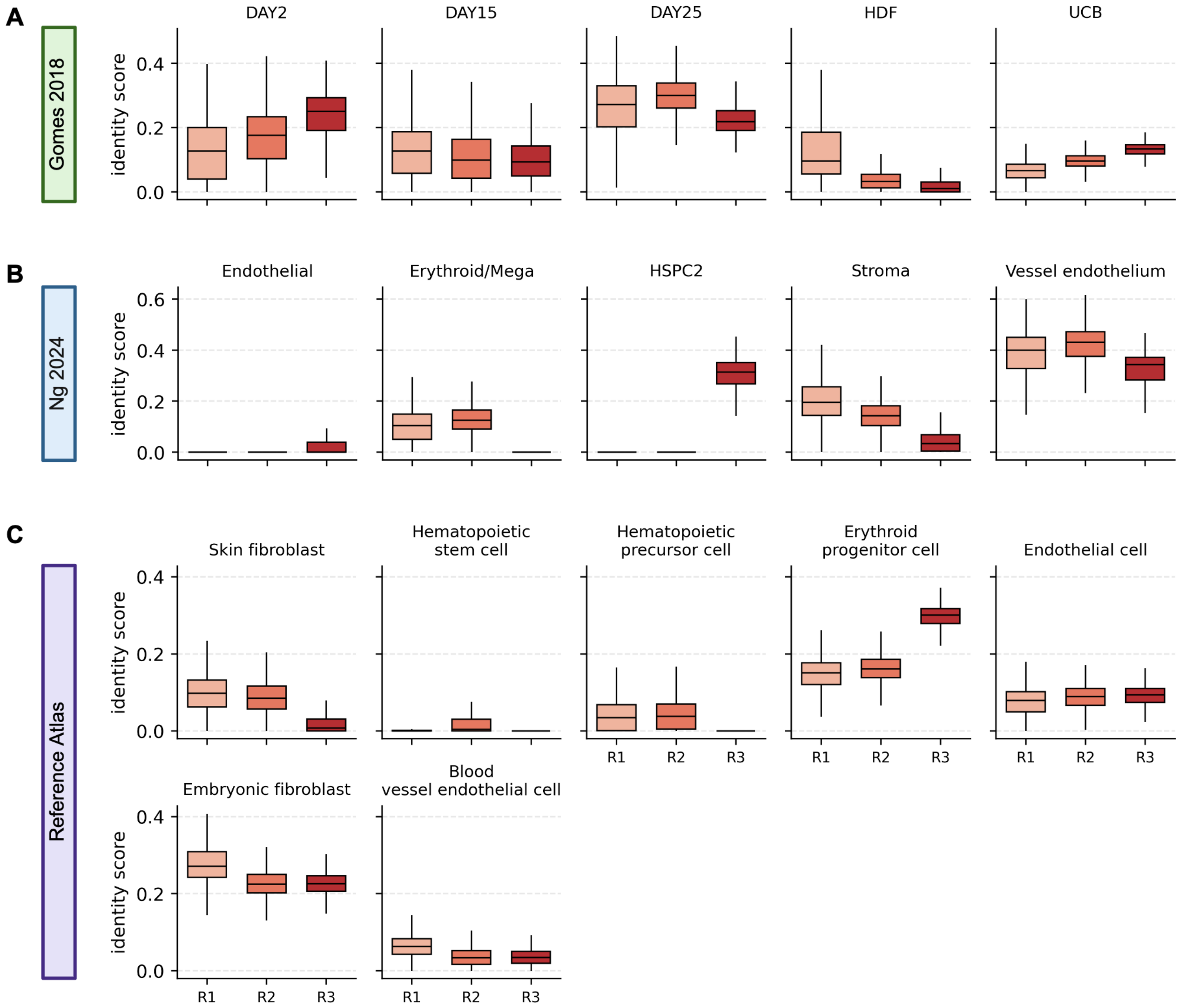

Figure S26: **Capybara identity scores on cell subtypes, related to Figure 4. (A)** Capybara identity scores of reprogrammed clusters to cell types in[9]. **(B)** Capybara identity scores of reprogrammed clusters to selected cell types in[8]. **(C)** Capybara identity scores of reprogrammed clusters to selected cell types in our native reference atlas. All data are represented as median and IQR, with whiskers extending to $1.5 \times$ IQR. Created in BioRender. Cwycyshyn, J. (2026) https://BioRender.com/ic2ueg4.

Supplementary Tables

Cell Type	Gene	logFC	Pct in Group	Pct in Ref
vascular endothelial cell	TM4SF1	5.77	0.90	0.18
vascular endothelial cell	MT2A	4.17	0.91	0.44
vascular endothelial cell	CD59	3.56	0.84	0.34
vascular endothelial cell	SOD2	3.95	0.83	0.35
vascular endothelial cell	UBC	2.14	0.94	0.74
vascular endothelial cell	EIF1	1.73	0.96	0.80
vascular endothelial cell	NFKBIA	2.83	0.88	0.53
vascular endothelial cell	IFI27	3.43	0.75	0.16
vascular endothelial cell	JUND	2.67	0.88	0.49
vascular endothelial cell	ADAMTS9	3.87	0.69	0.13
cord blood HSC	SNHG29	4.41	0.71	0.22
cord blood HSC	HNRNPA1	2.48	0.82	0.69
cord blood HSC	LDHB	3.34	0.76	0.51
cord blood HSC	EEF1B2	2.45	0.81	0.64
cord blood HSC	GAS5	4.20	0.68	0.22
cord blood HSC	BTF3	1.96	0.78	0.71
cord blood HSC	NPM1	1.98	0.77	0.69
cord blood HSC	AIF1	3.62	0.62	0.23
cord blood HSC	RPSA	1.53	0.78	0.73
cord blood HSC	RPL4	1.49	0.75	0.72
embryonic fibroblast	FN1	5.05	0.97	0.24
embryonic fibroblast	GPC3	5.55	0.95	0.14
embryonic fibroblast	COL3A1	5.71	0.97	0.24
embryonic fibroblast	EGFL6	7.62	0.87	0.03
embryonic fibroblast	DLK1	6.83	0.89	0.09
embryonic fibroblast	PLAGL1	4.71	0.91	0.20
embryonic fibroblast	SERPINE2	5.93	0.88	0.15
embryonic fibroblast	HGF	5.90	0.87	0.07
embryonic fibroblast	COL6A2	4.30	0.94	0.31
embryonic fibroblast	CDH11	4.71	0.89	0.14
endothelial cell	EMCN	3.94	0.63	0.10
endothelial cell	EGFL7	3.34	0.68	0.18
endothelial cell	EPAS1	3.12	0.68	0.21
endothelial cell	LDB2	3.51	0.65	0.16
endothelial cell	A2M	3.04	0.69	0.20
endothelial cell	FLT1	3.72	0.61	0.10
endothelial cell	PECAM1	3.20	0.64	0.19
endothelial cell	PTPRB	4.02	0.57	0.07
endothelial cell	ADGRL4	3.65	0.54	0.08
endothelial cell	TM4SF1	2.80	0.64	0.18
erythroid progenitor	H2AZ1	3.27	0.88	0.51
erythroid progenitor	HMGB1	2.58	0.88	0.74
erythroid progenitor	NPM1	2.60	0.89	0.69

Continued on next page

Table S1: **Top 10 DEGs in fibroblast, endothelial, and hematopoietic populations, related to Figure 1.**

Cell Type	Gene	logFC	Pct in Group	Pct in Ref
erythroid progenitor	STMN1	3.63	0.83	0.34
erythroid progenitor	ATP5IF1	3.54	0.81	0.38
erythroid progenitor	PRDX2	3.82	0.80	0.38
erythroid progenitor	BLVRB	4.16	0.76	0.26
erythroid progenitor	HINT1	2.43	0.85	0.64
erythroid progenitor	TUBA1B	2.89	0.88	0.59
erythroid progenitor	TYMS	4.43	0.65	0.09
fibroblast	DCN	4.44	0.85	0.23
fibroblast	COL6A2	3.05	0.79	0.25
fibroblast	COL1A2	3.55	0.77	0.22
fibroblast	COL6A1	2.98	0.72	0.20
fibroblast	LUM	3.88	0.66	0.13
fibroblast	SERPINF1	3.65	0.63	0.12
fibroblast	COL6A3	3.12	0.65	0.15
fibroblast	FBLN1	3.16	0.65	0.15
fibroblast	MEG3	2.94	0.67	0.21
fibroblast	FSTL1	2.74	0.68	0.21
multipotent progenitor (MPP)	SPINK2	6.52	0.92	0.04
multipotent progenitor (MPP)	HINT1	2.95	0.99	0.64
multipotent progenitor (MPP)	RACK1	2.85	1.00	0.73
multipotent progenitor (MPP)	AIF1	3.98	0.98	0.22
multipotent progenitor (MPP)	NPM1	2.75	1.00	0.69
multipotent progenitor (MPP)	RPLP1	2.52	1.00	0.83
multipotent progenitor (MPP)	HSP90AB1	2.69	1.00	0.70
multipotent progenitor (MPP)	MACROH2A1	3.01	0.93	0.37
multipotent progenitor (MPP)	ENO1	2.55	0.97	0.54
multipotent progenitor (MPP)	HOPX	4.01	0.79	0.09
hematopoietic precursor	MACROH2A1	2.50	0.88	0.37
hematopoietic precursor	RPS17	3.31	0.85	0.47
hematopoietic precursor	PRSS57	4.23	0.68	0.06
hematopoietic precursor	HNRNPA1	2.13	0.98	0.69
hematopoietic precursor	SEPTIN6	2.59	0.83	0.24
hematopoietic precursor	SERPINB1	2.70	0.82	0.29
hematopoietic precursor	GIHCG	3.64	0.66	0.10
hematopoietic precursor	RPS3	1.62	0.95	0.79
hematopoietic precursor	RPL3	1.72	0.98	0.78
hematopoietic precursor	RPS4X	1.65	0.95	0.78
hematopoietic stem cell (HSC)	SPINK2	4.88	0.74	0.05
hematopoietic stem cell (HSC)	SMIM24	4.90	0.70	0.04
hematopoietic stem cell (HSC)	PRSS57	4.50	0.71	0.05
hematopoietic stem cell (HSC)	RPS3	1.85	0.89	0.79
hematopoietic stem cell (HSC)	CYTL1	4.06	0.69	0.07
hematopoietic stem cell (HSC)	HMGA1	3.04	0.79	0.26
hematopoietic stem cell (HSC)	GIHCG	3.54	0.70	0.10

Continued on next page

Table S1: **Top 10 DEGs in fibroblast, endothelial, and hematopoietic populations, related to Figure 1.**

Cell Type	Gene	logFC	Pct in Group	Pct in Ref
hematopoietic stem cell (HSC)	EEF1B2	2.07	0.85	0.64
hematopoietic stem cell (HSC)	RPS24	1.48	0.86	0.78
hematopoietic stem cell (HSC)	RPS23	1.54	0.87	0.78
megakaryocyte progenitor	H3-3A	4.87	1.00	0.67
megakaryocyte progenitor	PTMA	3.66	1.00	0.82
megakaryocyte progenitor	TAGLN2	4.89	1.00	0.55
megakaryocyte progenitor	PLEK	7.53	0.95	0.15
megakaryocyte progenitor	CD164	5.48	0.98	0.44
megakaryocyte progenitor	CDK6	5.66	0.95	0.23
megakaryocyte progenitor	HMGB1	3.90	1.00	0.74
megakaryocyte progenitor	YBX1	3.89	0.99	0.69
megakaryocyte progenitor	ITGA2B	9.24	0.91	0.03
megakaryocyte progenitor	TALDO1	5.13	0.98	0.40
dermal fibroblast	DCN	5.84	0.97	0.29
dermal fibroblast	CXCL14	6.22	0.82	0.08
dermal fibroblast	COL6A2	4.05	0.90	0.30
dermal fibroblast	S100A6	3.50	0.98	0.64
dermal fibroblast	COL1A2	4.54	0.90	0.26
dermal fibroblast	C1S	4.25	0.83	0.18
dermal fibroblast	CTSK	4.89	0.78	0.11
dermal fibroblast	GSN	4.05	0.90	0.40
dermal fibroblast	SOD3	4.83	0.78	0.11
dermal fibroblast	COL6A1	3.92	0.84	0.24
venous endothelial cell	TM4SF1	5.34	0.90	0.19
venous endothelial cell	CD59	3.34	0.87	0.34
venous endothelial cell	HLA-E	2.43	0.94	0.61
venous endothelial cell	PLVAP	4.38	0.69	0.07
venous endothelial cell	IFI27	3.88	0.74	0.16
venous endothelial cell	GNG11	3.01	0.80	0.26
venous endothelial cell	IGFBP7	2.98	0.90	0.40
venous endothelial cell	ACKR1	6.27	0.62	0.04
venous endothelial cell	SPARCL1	3.34	0.81	0.32
venous endothelial cell	CAV1	3.06	0.79	0.25

Table S1: **Top 10 DEGs in fibroblast, endothelial, and hematopoietic populations, related to Figure 1.**

Cell Type	Gene	logFC	Pct in Group	Pct in Ref
vascular endothelial cell	CD59	3.56	0.84	0.34
vascular endothelial cell	JUND	2.67	0.88	0.49
vascular endothelial cell	ETS2	2.78	0.71	0.27
vascular endothelial cell	MAFF	2.91	0.65	0.19
vascular endothelial cell	TCF4	2.07	0.79	0.40
vascular endothelial cell	ATF3	2.70	0.62	0.21
vascular endothelial cell	KLF6	1.87	0.80	0.53
vascular endothelial cell	ETS1	2.10	0.64	0.27
vascular endothelial cell	SOX17	4.13	0.45	0.04
vascular endothelial cell	YWHAE	1.46	0.76	0.49
cord blood HSC	HNRNPA1	2.48	0.82	0.69
cord blood HSC	UBB	1.67	0.69	0.68
cord blood HSC	ENO1	1.98	0.58	0.55
cord blood HSC	RPS10	1.36	0.55	0.58
cord blood HSC	HMGA1	2.14	0.37	0.26
cord blood HSC	RAN	1.37	0.47	0.54
cord blood HSC	SOX4	1.13	0.31	0.34
cord blood HSC	NME1	1.74	0.22	0.21
cord blood HSC	TMSB4XP8	2.53	0.02	0.01
cord blood HSC	XRCC1	1.30	0.07	0.10
embryonic fibroblast	PLAGL1	4.71	0.91	0.20
embryonic fibroblast	PITX2	7.80	0.79	0.01
embryonic fibroblast	TCF21	3.53	0.62	0.08
embryonic fibroblast	PEG3	4.66	0.54	0.05
embryonic fibroblast	PDLIM5	2.85	0.69	0.23
embryonic fibroblast	HAND2	4.95	0.51	0.03
embryonic fibroblast	LIN28B	5.97	0.49	0.02
embryonic fibroblast	EZR	2.14	0.71	0.28
embryonic fibroblast	EPAS1	2.03	0.72	0.22
embryonic fibroblast	CREB3L1	3.64	0.53	0.06
endothelial cell	EPAS1	3.12	0.68	0.21
endothelial cell	ERG	3.26	0.47	0.09
endothelial cell	TCF4	1.82	0.69	0.40
endothelial cell	NFIB	1.78	0.57	0.27
endothelial cell	MECOM	3.29	0.39	0.09
endothelial cell	NPDC1	2.36	0.44	0.15
endothelial cell	MEF2C	1.66	0.56	0.27
endothelial cell	ID1	2.01	0.50	0.22
endothelial cell	ELK3	2.03	0.46	0.18
endothelial cell	ETS2	1.79	0.51	0.27
erythroid progenitor	HMGB1	2.58	0.88	0.74
erythroid progenitor	NFE2	4.40	0.55	0.06
erythroid progenitor	HMGA1	2.85	0.60	0.26
erythroid progenitor	RAN	1.89	0.69	0.53

Continued on next page

Table S2: **Top 10 differentially expressed TFs in fibroblast, endothelial, and hematopoietic populations, related to Figure 1.**

Cell Type	Gene	logFC	Pct in Group	Pct in Ref
erythroid progenitor	LMO2	3.44	0.52	0.14
erythroid progenitor	NME1	2.89	0.55	0.20
erythroid progenitor	KLF1	4.66	0.42	0.03
erythroid progenitor	GATA1	4.67	0.41	0.03
erythroid progenitor	ENO1	1.55	0.71	0.54
erythroid progenitor	HMGB2	1.96	0.57	0.35
fibroblast	PRRX1	2.93	0.51	0.10
fibroblast	ARID5B	1.88	0.62	0.31
fibroblast	NFIA	1.64	0.61	0.29
fibroblast	EGR1	1.78	0.55	0.27
fibroblast	ZBTB20	1.40	0.59	0.34
fibroblast	TWIST2	2.61	0.32	0.06
fibroblast	CEBPD	1.39	0.57	0.33
fibroblast	TCF4	1.10	0.63	0.39
fibroblast	PLAGL1	1.79	0.41	0.18
fibroblast	RBFOX2	1.41	0.42	0.17
multipotent progenitor (MPP)	ENO1	2.55	0.97	0.54
multipotent progenitor (MPP)	HMGB1	2.10	0.99	0.74
multipotent progenitor (MPP)	MSI2	2.95	0.79	0.20
multipotent progenitor (MPP)	HMGA1	2.89	0.79	0.25
multipotent progenitor (MPP)	LMO2	3.35	0.72	0.13
multipotent progenitor (MPP)	YBX1	1.83	0.98	0.68
multipotent progenitor (MPP)	HHEX	3.25	0.64	0.10
multipotent progenitor (MPP)	BCL11A	3.27	0.61	0.08
multipotent progenitor (MPP)	SOX4	2.04	0.85	0.33
multipotent progenitor (MPP)	APEX1	1.92	0.76	0.34
hematopoietic precursor	HNRNPA1	2.13	0.98	0.69
hematopoietic precursor	RPS4X	1.65	0.95	0.78
hematopoietic precursor	HMGA1	2.29	0.80	0.26
hematopoietic precursor	SOX4	2.09	0.85	0.34
hematopoietic precursor	RPL35	1.18	0.87	0.76
hematopoietic precursor	APEX1	1.74	0.75	0.34
hematopoietic precursor	NAP1L1	1.41	0.92	0.61
hematopoietic precursor	ENO1	1.56	0.88	0.55
hematopoietic precursor	LYL1	2.68	0.52	0.10
hematopoietic precursor	RPL6	1.21	0.93	0.76
hematopoietic stem cell (HSC)	HMGA1	3.04	0.79	0.26
hematopoietic stem cell (HSC)	RPS4X	1.58	0.87	0.78
hematopoietic stem cell (HSC)	APEX1	2.14	0.79	0.34
hematopoietic stem cell (HSC)	HNRNPA1	1.79	0.87	0.69
hematopoietic stem cell (HSC)	PARP1	2.07	0.71	0.23
hematopoietic stem cell (HSC)	ENO1	1.73	0.83	0.54
hematopoietic stem cell (HSC)	BCL11A	2.94	0.55	0.09
hematopoietic stem cell (HSC)	MYB	3.62	0.50	0.05
hematopoietic stem cell (HSC)	MSI2	2.18	0.67	0.20

Continued on next page

Table S2: **Top 10 differentially expressed TFs in fibroblast, endothelial, and hematopoietic populations, related to Figure 1.**

Cell Type	Gene	logFC	Pct in Group	Pct in Ref
hematopoietic stem cell (HSC)	NFE2	2.52	0.50	0.07
megakaryocyte progenitor	TAGLN2	4.89	1.00	0.55
megakaryocyte progenitor	HMGB1	3.90	1.00	0.74
megakaryocyte progenitor	YBX1	3.89	0.99	0.69
megakaryocyte progenitor	LMO2	6.06	0.94	0.15
megakaryocyte progenitor	ENO1	4.09	0.99	0.55
megakaryocyte progenitor	NFE2	6.15	0.82	0.07
megakaryocyte progenitor	TAL1	5.94	0.79	0.06
megakaryocyte progenitor	XBP1	3.44	0.89	0.39
megakaryocyte progenitor	ETV6	3.70	0.82	0.22
megakaryocyte progenitor	MSI2	3.37	0.72	0.21
dermal fibroblast	ANXA1	2.30	0.81	0.41
dermal fibroblast	PRRX1	2.84	0.56	0.14
dermal fibroblast	TWIST1	3.23	0.40	0.07
dermal fibroblast	PRNP	1.93	0.53	0.25
dermal fibroblast	PRRX2	3.80	0.35	0.04
dermal fibroblast	OSR2	3.44	0.36	0.05
dermal fibroblast	NFIX	2.51	0.40	0.12
dermal fibroblast	RBM3	1.16	0.69	0.53
dermal fibroblast	XG	5.06	0.31	0.01
dermal fibroblast	KLF4	1.69	0.49	0.24
venous endothelial cell	CD59	3.34	0.87	0.34
venous endothelial cell	ETS2	2.66	0.73	0.27
venous endothelial cell	NPDC1	3.11	0.64	0.15
venous endothelial cell	ZNF385D	3.13	0.57	0.10
venous endothelial cell	EPAS1	2.44	0.66	0.22
venous endothelial cell	TCF4	1.85	0.79	0.40
venous endothelial cell	ELK3	2.28	0.57	0.19
venous endothelial cell	ID1	2.43	0.59	0.22
venous endothelial cell	SOX7	3.62	0.42	0.05
venous endothelial cell	TAGLN2	1.32	0.82	0.55

Table S2: **Top 10 differentially expressed TFs in fibroblast, endothelial, and hematopoietic populations, related to Figure 1.**

Gene	t	df	$\bar{x}_A \pm SD$	$\bar{x}_B \pm SD$	Mean diff	95% CI	Effect size	P value
GATA2	25.37	2.46	-9.87 ± 0.76	2.42 ± 0.26	11.69	10.02–13.35	$\eta^2 = 0.996$	0.0005
GFI1B	9.98	2.39	-10.64 ± 1.00	-4.59 ± 0.32	6.06	3.82–8.29	$\eta^2 = 0.977$	0.0053
FOS	61.33	3.53	-13.69 ± 0.39	3.04 ± 0.27	16.73	15.93–17.53	$\eta^2 = 0.999$	< 0.0001
STAT5A	25.27	2.19	-10.08 ± 0.94	4.21 ± 0.21	14.28	12.07–16.49	$\eta^2 = 0.997$	0.0009
REL	17.27	3.98	-10.16 ± 0.57	-1.90 ± 0.61	8.26	6.93–9.59	$\eta^2 = 0.987$	< 0.0001

Table S3: **Statistical details for qRT-PCR analyses, related to Figure 2.** Group comparisons were performed using unpaired two-tailed t-tests with Welch's correction. Effect size is reported as η^2 (eta squared). Sample means and standard deviations are reported as $\bar{x} \pm SD$ for control (A) and treatment (B). Mean differences are reported as $B - A$ with 95% confidence intervals.

Barcoded Phase	Predicted G1	Predicted G2M	Predicted S
G1	2708 (47.5%)	1008 (17.7%)	1151 (20.2%)
G2M	87 (6.9%)	910 (72.1%)	246 (19.5%)
S	331 (26.3%)	478 (38.0%)	563 (44.8%)

Table S4: **Confusion matrix of predicted cell cycle phases vs. feature barcodes, related to Figure 3.** Raw counts and row-normalized percentages are shown.

Cluster	Annotation	Data Key	Count	Proportion
A1	R2	This Study	3924	0.281000
A1	hematopoietic multipotent progenitor cell	Reference	2736	0.196000
A1	HSC	This Study	1343	0.096000
A1	hematopoietic stem cell	Reference	1142	0.082000
A1	R1	This Study	1004	0.072000
A10	mesothelial cell	Reference	4419	0.402000
A10	mesenchymal cell	Reference	2810	0.256000
A10	hematopoietic multipotent progenitor cell	Reference	1108	0.101000
A10	fibroblast of lung	Reference	746	0.068000
A10	pulmonary interstitial fibroblast	Reference	404	0.037000
A11	fibroblast	Reference	2843	0.312000
A11	mesenchymal cell	Reference	1059	0.116000
A11	fibroblast of cardiac tissue	Reference	979	0.107000
A11	initial	This Study	704	0.077000
A11	fibro/adipogenic progenitor cell	Reference	659	0.072000
A12	HSPC1	Ng 2024	12965	0.369000
A12	EMK Pro1	Ng 2024	3502	0.100000
A12	HSPC2	Ng 2024	3394	0.097000
A12	Mye Pro2	Ng 2024	3366	0.096000
A12	EMK Pro2	Ng 2024	3289	0.094000
A13	endothelial cell of lymphatic vessel	Reference	3237	0.734000
A13	CLP	This Study	453	0.103000
A13	lymphoid lineage restricted progenitor cell	Reference	353	0.080000
A13	endothelial cell	Reference	241	0.055000
A13	early lymphoid progenitor	Reference	17	0.004000
A14	Venous	Ng 2024	7742	0.878000
A14	endothelial cell	Reference	378	0.043000
A14	capillary endothelial cell	Reference	71	0.008000
A14	Endo	Ng 2024	53	0.006000
A14	Hemogenic	Ng 2024	48	0.005000
A15	HSPC1	Ng 2024	4610	0.336000
A15	Hemogenic	Ng 2024	4413	0.322000
A15	HSPC2	Ng 2024	1708	0.124000
A15	EMK Pro1	Ng 2024	1061	0.077000
A15	EMK Pro2	Ng 2024	549	0.040000

Continued on next page

Table S5: **Top 5 annotation-data key pairs by cluster, related to Figure 4.**

Cluster	Annotation	Data Key	Count	Proportion
A16	DAY25	Gomes 2018	91	0.453000
A16	DAY2	Gomes 2018	38	0.189000
A16	HDF	Gomes 2018	37	0.184000
A16	DAY15	Gomes 2018	35	0.174000
A2	EMK Pro1	Ng 2024	12141	0.206000
A2	Mega	Ng 2024	11511	0.195000
A2	EMK Pro2	Ng 2024	8835	0.150000
A2	Eos	Ng 2024	8556	0.145000
A2	Gran	Ng 2024	5133	0.087000
A3	fibroblast	Reference	6224	0.213000
A3	Mono	Ng 2024	4597	0.157000
A3	Gran	Ng 2024	4430	0.151000
A3	Mye Pro1	Ng 2024	4148	0.142000
A3	fibroblast of mammary gland	Reference	3671	0.125000
A4	mesenchymal cell	Reference	7741	0.218000
A4	initial	This Study	6564	0.185000
A4	fibroblast of mammary gland	Reference	4874	0.137000
A4	fibroblast	Reference	3204	0.090000
A4	HSPC1	Ng 2024	2792	0.079000
A5	adipocyte	Reference	4000	0.716000
A5	endothelial tip cell	Reference	1060	0.190000
A5	fibroblast of mammary gland	Reference	109	0.020000
A5	capillary endothelial cell	Reference	104	0.019000
A5	HSPC3	Ng 2024	46	0.008000
A6	Mye Pro1	Ng 2024	13693	0.244000
A6	HSPC1	Ng 2024	12154	0.217000
A6	Mye Pro2	Ng 2024	10104	0.180000
A6	Mono	Ng 2024	5162	0.092000
A6	HSPC2	Ng 2024	3704	0.066000
A7	fibroblast	Reference	13892	0.215000
A7	Strom2	Ng 2024	13569	0.210000
A7	Strom3	Ng 2024	12507	0.194000
A7	mesenchymal cell	Reference	9202	0.143000
A7	Strom1	Ng 2024	2854	0.044000
A8	pericyte	Reference	7808	0.442000
A8	vascular associated smooth muscle cell	Reference	4877	0.276000
A8	fibroblast	Reference	1937	0.110000
A8	mural cell	Reference	1420	0.080000
A8	lung pericyte	Reference	294	0.017000
A9	Art1	Ng 2024	8402	0.150000
A9	endothelial cell	Reference	7738	0.138000
A9	blood vessel endothelial cell	Reference	7446	0.133000
A9	Endo	Ng 2024	6468	0.115000
A9	vein endothelial cell	Reference	5100	0.091000

Table S5: **Top 5 annotation-data key pairs by cluster, related to Figure 4.**

Dataset	Cells	Genes	Cell types	Accession	Citation
Gomes 2018	286	13,492	5	GEO: GSE51025	Gomes et al.[9]
Pellin 2019	21,412	13,979	11	GEO: GSE117498	Pellin et al.[12]
Setty 2019	5,780	10,438	8	N/A	Setty et al.[7]
Georgolopoulos 2021	53,511	13,384	7	GEO: GSE182816	Georgolopoulos et al.[11]
Weng 2024	25,252	14,179	20	GEO: GSE219015	Weng et al.[13]
Ng 2024	252,607	15,023	20	GEO: GSE232710	Ng et al.[8]

Table S6: **Summary of Single-Cell Datasets, related to Figure 4.** Setty 2019 data is available via the Human Cell Atlas portal (https://explore.data.humancellatlas.org/projects/091cf39b-01bc-42e5-9437-f419a66c8a45).

FH3	FH4a	FH6
GATA2<br>GFI1B<br>FOS	GATA2<br>GFI1B<br>FOS<br>REL<br>STAT5A	GATA2<br>GFI1B<br>FOS<br>REL<br>STAT5A
**Vector preparation**		
individual lenti per TF	single lenti	individual lenti per TF

Table S7: **TF recipes and lentiviral vector preparation methods, related to STAR Methods.**

**Primers**	
GATA2-V5 Fwd	TTCGAGGAGCTGTCAAAGTG
GFI1B-V5 Fwd	CCAGAAGTCCGACATGAAGAAG
FOS-V5 Fwd	GGACCTATCTGGGTCCTTCTA
STAT5A-V5 Fwd	CAAGTGGTCCCTGAGTTTGT
REL-V5 Fwd	TCCATGCCATCAGCAGATTTA
Common-V5 Rev	CTAGGAGTGGGTTTGGGATTG
LDLR Fwd	ACCGACTCTGTCCTGGGCACTG
LDLR Rev	AGTTCCCCAGTCAGTCCAGTA

Table S8: **Primers used for qRT-PCR validation of 5TF expression, related to STAR Methods.**

Dataset	Reprogram	Control fibroblasts	Bone marrow HSPCs	Reprogram time-series
**Name**	Library 1	Library 2	Library 3	Time-series
**Barcoding technology**	10x Genomics	10x Genomics	10x Genomics	Split-pool combinatorial barcoding
**Multiplexing method**	N/A	TotalSeqB hashtag antibodies	TotalSeqB hashtag antibodies	Split-pool combinatorial barcoding
**Cell populations**	CD34+ D48 CD34- D48	Fibroblasts (G1) Fibroblasts (S) Fibroblasts (G2M)	CD34+/CD38- CD34+/CD38+	Control fibroblasts; D0, D7, D14, D21
**Total cells**	8,357	6,960	5,608	6,174
**Mean counts/cell**	12,143	14,571	21,013	5,416
**Median counts/cell**	11,091	14,160	18,366	4,511
**Mean genes/cell**	3,606	3,694	4,493	2,684
**Median genes/cell**	3,576	3,653	4,417	2,521

Table S9: **Overview of scRNA-seq libraries generated in this study, related to Figures $2-4$ and STAR Methods.** Total cells and summary statistics are post-quality control.

10x Genomics

Next GEM Single Cell 3' Kit (V3.1,V4)

Parse Biosciences

Parse Cell Fixation Kit v3
Evercode WT Mini v3

ONT

**Sequencer**	**3' Protocol Version**	**Adapter Kit**
GridION	SST_V9148_V111_REVB_12JAN2022	cDNA-PCR Sequencing Kit (SQK-PCS111)
PromethION P2 Solo	SST_V9198_V114_REVB_06DEC2023	Ligation Sequencing Kit V14 (SQK-LSK114)
	**Parse Protocol Version**	**Adapter Kit**
PromethION P2 Solo	SST_9198_V114_REVK_29JAN2025	Ligation Sequencing Kit V14 (SQK-LSK114)

Primers

Transcript libraries		
Fwd: 5' - [Biotin] -	CAGCACTTGCCTGTCGCTCTATCTTCCTACACGACGCTCTTCCGATCT	- 3'
Rev: 5' -	CAGCTTTCTGTTGGTGCTGATATTGCAAGCAGTGGTATCAACGCAGAG	- 3'
Hashing library		
Fwd: 5' - [Biotin] -	GCAGCGTCAGATGTGTATAAGAGACAG	- 3'
Fwd: 5' -	GTGACTGGAGTTCAGACGT	- 3'

Table S10: **Protocols and primers used for single cell barcoding and ONT sequencing, related to STAR Methods.**

Method	Variant	Description
PCA	-	Unintegrated baseline
Harmony	Batch only	Correct batch effects
Harmony	Batch + cell cycle	Regress cell cycle, then correct batch
scVI	Batch only	Batch as covariate
scVI	Batch + cell cycle	Batch and cell cycle as covariates
scANVI	Semi-supervised	Adds cell type label guidance

Table S11: **Summary of data integration strategies evaluated for pseudotime projection, related to STAR Methods.**

Code	Description
SE	Skipping exon
A5	Alternative 5′ splice site
A3	Alternative 3′ splice site
MX	Mutually exclusive exon
RI	Retained intron
AF	Alternative first exon
AL	Alternative last exon

Table S12: **Alternative splicing event types, related to STAR Methods.**

References

1. THE TABULA SAPIENS CONSORTIUM. The Tabula Sapiens: A Multiple-Organ, Single-Cell Transcriptomic Atlas of Humans. Science 2022; 376:eabl4896
2. Franzén O, Gan LM, and Björkegren JLM. PanglaoDB: A Web Server for Exploration of Mouse and Human Single-Cell RNA Sequencing Data. Database 2019; 2019:baz046
3. Tirosh I, Venteicher AS, Hebert C, Escalante LE, Patel AP, Yizhak K, Fisher JM, Rodman C, Mount C, Filbin MG, et al. Single-Cell RNA-seq Supports a Developmental Hierarchy in Human Oligodendroglioma. Nature 2016; 539:309–13
4. Korsunsky I, Millard N, Fan J, Slowikowski K, Zhang F, Wei K, Baglaenko Y, Brenner M, Loh Pr, and Raychaudhuri S. Fast, Sensitive and Accurate Integration of Single-Cell Data with Harmony. Nat Methods 2019; 16:1289–96
5. Lopez R, Regier J, Cole MB, Jordan MI, and Yosef N. Deep Generative Modeling for Single-Cell Transcriptomics. Nat Methods 2018; 15:1053–8
6. Xu C, Lopez R, Mehlman E, Regier J, Jordan MI, and Yosef N. Probabilistic Harmonization and Annotation of Single-cell Transcriptomics Data with Deep Generative Models. Molecular Systems Biology 2021; 17:e9620
7. Setty M, Kiseliovas V, Levine J, Gayoso A, Mazutis L, and Pe'er D. Characterization of Cell Fate Probabilities in Single-Cell Data with Palantir. Nat Biotechnol 2019; 37:451–60
8. Ng ES, Sarila G, Li JY, Edirisinghe HS, Saxena R, Sun S, Bruveris FF, Labonne T, Sleebs N, Maytum A, et al. Long-Term Engrafting Multilineage Hematopoietic Cells Differentiated from Human Induced Pluripotent Stem Cells. Nat Biotechnol 2025; 43:1274–87

9. Gomes AM, Kurochkin I, Chang B, Daniel M, Law K, Satija N, Lachmann A, Wang Z, Ferreira L, Ma'ayan A, et al. Cooperative Transcription Factor Induction Mediates Hemogenic Reprogramming. Cell Reports 2018; 25:2821–2835.e7
10. Wolf FA, Hamey FK, Plass M, Solana J, Dahlin JS, Göttgens B, Rajewsky N, Simon L, and Theis FJ. PAGA: Graph Abstraction Reconciles Clustering with Trajectory Inference through a Topology Preserving Map of Single Cells. Genome Biology 2019; 20:59
11. Georgolopoulos G, Psatha N, Iwata M, Nishida A, Som T, Yiangou M, Stamatoyannopoulos JA, and Vierstra J. Discrete Regulatory Modules Instruct Hematopoietic Lineage Commitment and Differentiation. Nat Commun 2021; 12:6790
12. Pellin D, Loperfido M, Baricordi C, Wolock SL, Montepeloso A, Weinberg OK, Biffi A, Klein AM, and Biasco L. A Comprehensive Single Cell Transcriptional Landscape of Human Hematopoietic Progenitors. Nat Commun 2019; 10:2395
13. Weng C, Yu F, Yang D, Poeschla M, Liggett LA, Jones MG, Qiu X, Wahlster L, Caulier A, Hussmann JA, et al. Deciphering Cell States and Genealogies of Human Haematopoiesis. Nature 2024; 627:389–98
